# Affordable, portable and self-administrable electrical impedance tomography enables global and regional lung function assessment

**DOI:** 10.1038/s41598-022-24330-2

**Published:** 2022-11-30

**Authors:** Fedi Zouari, Wei Yi Oon, Dipyaman Modak, Wing Hang Lee, Wang Chun Kwok, Peng Cao, Wei-Ning Lee, Terence Chi Chun Tam, Eddie C. Wong, Russell W. Chan

**Affiliations:** 1 Gense Technologies Ltd., Hong Kong, China; 2 Department of Medicine, Queen Mary Hospital, Hong Kong, China; 3 Department of Medicine, Li Ka Shing Faculty of Medicine, The University of Hong Kong, Hong Kong, China; 4 Department of Diagnostic Radiology, Li Ka Shing Faculty of Medicine, The University of Hong Kong, Hong Kong, China; 5 Department of Electrical and Electronic Engineering, Faculty of Engineering, The University of Hong Kong, Hong Kong, China

**Keywords:** Medical research, Biomarkers, Biomedical engineering, Electrical and electronic engineering

## Abstract

Accessibility of diagnostic screening and treatment monitoring devices for respiratory diseases is critical in promoting healthcare and reducing sudden complications and mortality. Spirometry is the standard for diagnosing and monitoring several lung diseases. However, it lacks regional assessment capabilities necessary for detecting subtle regional changes in certain diseases. It also requires challenging breathing maneuvers difficult for elderlies, children, and diseased patients. Here, we actualized an affordable, portable, and self-administrable electrical impedance tomography (EIT) system for home-based lung function assessment and telemedicine. Through simultaneous EIT-spirometry trials on healthy subjects, we demonstrated that our device can predict spirometry indicators over a wide range and can provide regional mapping of these indicators. We further developed a close-to-effortless breathing paradigm and tested it by longitudinally monitoring a COVID-19 discharged subject and two healthy controls with results suggesting the paradigm can detect initial deterioration followed by recovery. Overall, the EIT system can be widely applicable for lung function screening and monitoring both at homes and clinics.

## Main

Respiratory diseases encompass five of the thirty most common causes of severe illness and death worldwide^1^. Diagnostic screening and continuous monitoring of these diseases are critical for improving patients’ healthcare, and reducing sudden complications and mortality. While home-based screening and monitoring is a practical and cost-effective way to alleviate the burden associated with these disease^2–4^, its effectiveness is often challenged because of the lack of self-administrable, home-based and standard medical tools^5,6^.

To date, spirometry^7^ is the standard lung function assessment to evaluate overall aerodynamics, identify and monitor different conditions including COVID-19^8^. However, spirometry lacks the capability for regional assessment which is necessary for detecting, assessing and monitoring regional changes in certain lung diseases, such as chronic obstructive pulmonary disease (COPD)^9^ and potentially long COVID-19^10^. For instance, spirometry missed 10.4% of patients with COPD who have significant emphysema^9^. Furthermore, only 9% of COVID-19 discharged patients had no residual lung computed tomography (CT) abnormalities^10^ months after discharge, even though spirometry indices were all normal^10^. These studies signify the need for regional assessment of pulmonary functions.

Regional lung function is typically assessed through CT, hyperpolarized magnetic resonance imaging (MRI) or nuclear imaging methods^11^. However, these techniques are typically costly and have poor temporal resolution, which may not be ideal for lung function monitoring. Electrical impedance tomography^12,13^ (EIT) is an increasingly used radiation-free and non-invasive biomedical imaging technique for monitoring lung function, particularly infants in intensive care units^14,15^. Although EIT has low spatial resolution, its high temporal resolution is suitable for lung function testing. As such, recent research efforts were allocated to establish a relation between EIT and standard lung function measures to facilitate the interpretability of EIT results. For instance, a previous study developed a parametric model of the relationship between lung volume change, EIT conductivity change and anthropometric information^16^. However, they did not apply standard spirometry breathing maneuvers, as such whether EIT can directly reflect spirometry indicators remains inconclusive. More recently, this relationship has been investigated^17^, yet subject-wise calibration is required to enable the prediction of spirometry indicators from EIT. Overall, the use of EIT as a standalone method to predict standard lung function spirometry indicators is yet to be demonstrated.

Despite the recent advances in electronics which enabled the development of portable EIT devices^18,19^, commercial EIT systems remain costly, bulky and require clinicians to operate and interpret^13^. Hence, the wide usability of EIT systems on diagnostic screening of lung diseases and telemedicine applications of monitoring pulmonary functions is still hindered for both home-based or clinical settings. Here, we designed and implemented an affordable, portable, and self-administrable EIT system and established its association with standard spirometry. Our portable EIT system not only can be used as a standalone device to predict global spirometry indicators without subject-wise calibration (an advantage over existing EIT systems), but also provides regional mapping of these indicators (an advantage over spirometry). Furthermore, since spirometry requires subjects to perform a series of maneuvers correctly, which is difficult for elderly, children, and patients with severe lung impairments, we developed a novel close-to-effortless guided breathing paradigm for home-based lung function assessment. We demonstrated that this paradigm could reflect global and regional lung functions over healthy subjects. We further assessed the paradigm by longitudinally monitoring a COVID-19 discharged subject and two controls, with results suggesting initial deterioration followed by recovery for the COVID-19 discharged subject. It should be noted that the proposed close-to-effortless breathing paradigm is not intended to replace spirometry paradigm. Instead, it can be used as an alternative for screening and monitoring, which should be easier compared to the gold standard spirometry paradigm since it requires subjects to perform a series of manoeuvres correctly.

## Results

### Design of home-based lung function assessment system for telemedicine

**Figure 1:**
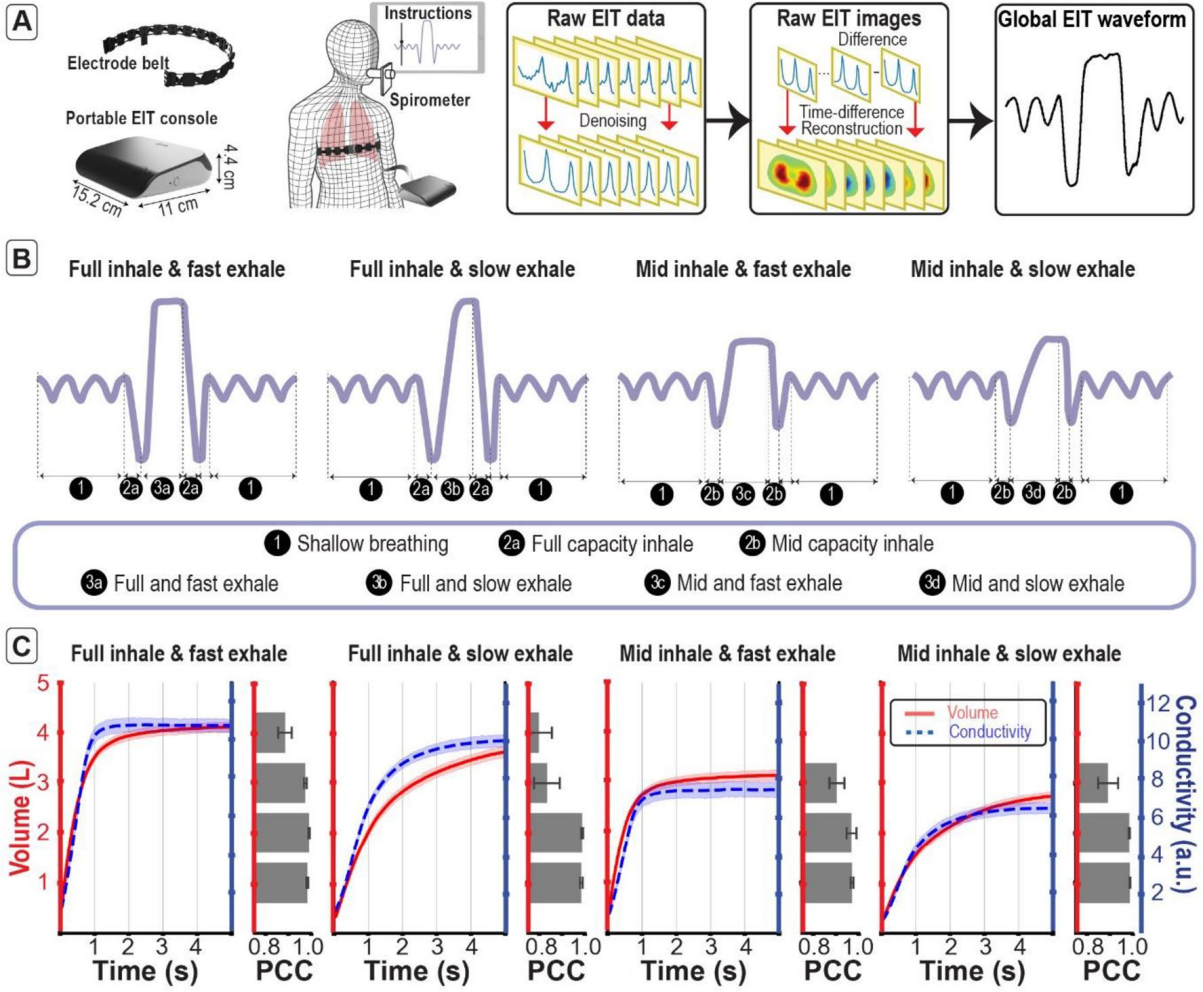
The portable EIT system and its data processing pipelines extracts conductivity-time curves that are highly correlated with the volume-time curves under different breathing paradigms. (**A**) The EIT system consists of a portable console, electrode belt, mobile app interface, and cloud-based processing pipeline. Raw EIT data is first acquired followed by denoising, time-difference EIT image reconstruction, and global conductivity waveform extraction. (**B**) Simultaneous EIT and spirometry were applied with four different breathing paradigms, including a combination of full or mid capacity inhale, and fast or slow exhale. (**C**) The mean (± standard error) of the extracted global conductivity-time curve and the volume-time curve agree as shown by the bar plots of the Pearson’s correlation coefficient (PCC; ± standard error) computed on the volume segments 0-1L, 1-2L, 2-3L, and 3-4L. The drawings of the electrode belt and the portable EIT console were made using Dassault Systemes Solidworks 2020, Luxion Keyshot 9 and Adobe Photoshop CC 2019. The drawings of the dummy person and spirometer were made using Adobe Illustrator 2021.

To support home-based screening and monitoring, we designed the EIT system taking into consideration the portability, self-administrability and cost-effectiveness. We developed a palm-size and light-weight console with dimensions of 15.2 × 11.0 × 4.4 cm^3^ and < 300 g (Fig. 1A). The portable console functions together with a reusable and disinfectable 16-channel electrode belt (Fig. 1A), mobile app interface, and cloud-based processing pipeline. The portability function is supported through the integration of a power and battery management module enabling the complete system to operate with a constant power supply of 3.3 V, thus it can function either through a power socket or a Li-ion battery. Note that several EIT systems^20–22^ require a higher power supply ranging from ± 5 V (total of 10 V) to ± 15 V (total of 30 V). To enable the portability of these systems, it is necessary to use multiple batteries which is more costly and impose additional challenges in cooling the system and fulfilling the International Electrotechnical Commission (IEC) regulations. The developed system also ensures users’ safety by limiting the generated current amplitude up to 1 mApp in accordance with regulatory guidelines of medical devices issued by the IEC (console details see Supplementary Figure S1 and online methods). The developed system can achieve flexible frame rate up to 50 frames per second through a triple interleaved ADC method (fps), enabling the detection of high frequency conductivity changes such as the ones during forced exhalation in spirometry tests (more details in online methods). The electrode belt consists of an elastic silicon band with sixteen equally spaced disposable carbon gel electrodes and come in sizes ranging from 65 to 120 cm in length with extendable range of 10% of its original length to accommodate chest circumference variance between populations and different breathing maneuvers. It is recommended to replace the gel electrodes every time after each use to ensure the best performance. The self-administrability function is supported through a mobile app which guides users to connect the console (see Supplementary Guide to connect to the console) and wear the electrode belt (see Supplementary Guide to wear the lung belt) and instructs users to perform breathing paradigms (Supplementary Guide to perform the lung test and Supplementary Figure S2). The acquired data can be either processed in a cloud-based processing pipeline or through a desktop application. The acquired raw data is denoised, then the time-difference EIT images are reconstructed and processed to generate functional maps, where the EIT-derived indicators are further extracted (Supplementary Figure S3). Streamlined data processing through a desktop or a cloud-based application enables both online and offline usability of the device while minimizing the additional cost for optimizing computational power of the console.

### Device characterization and performance validation

To enable our EIT system to benefit from the extensive spirometry literature, we aimed to establish the relation between the change in conductivity measured by EIT and the change in volume measured by spirometers. For this purpose, we applied EIT and spirometry (MIR Spirobank) simultaneously to fourteen healthy subjects of different age, height, weight, and ethnicity and predicted FVC (demographic and anthropometric details are in Supplementary Table S1). The predicted FVC of the selected subjects ranges from from 3.25 to 5.5 L with a mean 4.5L and a standard deviation of 0.8 L, as computed from the National Health and Nutrition Survey (NHANES) III^23^. All subjects in this work, unless otherwise stated, performed the tests in standing position and placed the belt at the thorax around the T4 and T5 vertebrae, that is right below the nipples for men and right below the breast for women. Each subject performed at least five repetitions of four customized breathing paradigms shown in Fig. 1B denoted (1) full inhale and fast exhale, (2) full inhale and slow exhale, (3) mid inhale and fast exhale; and (4) mid inhale and slow exhale. Note the first paradigm, i.e., full inhale and fast exhale, is the standard spirometry breathing paradigm. These paradigms were chosen in order to simulate a wide range of flow-rate and volume changes from different subjects (details see online methods).

The global conductivity waveforms measured with EIT and the volume-time waveforms measured with spirometer during the middle exhale section are extracted and shown in Fig. 1C. The volume and conductivity curves change in similar fashion for all breathing modes qualitatively. The Pearson’s correlation coefficient (PCC) computed between volume and conductivity on volume segments 0–1 L (L), 1–2 L, 2–3 L, and 3–4 L for each individual test is larger than 0.8 (*p* < 0.001, Fig. 1C), confirming that EIT can capture conductivity changes synchronously with volume changes.

**Figure 2:**
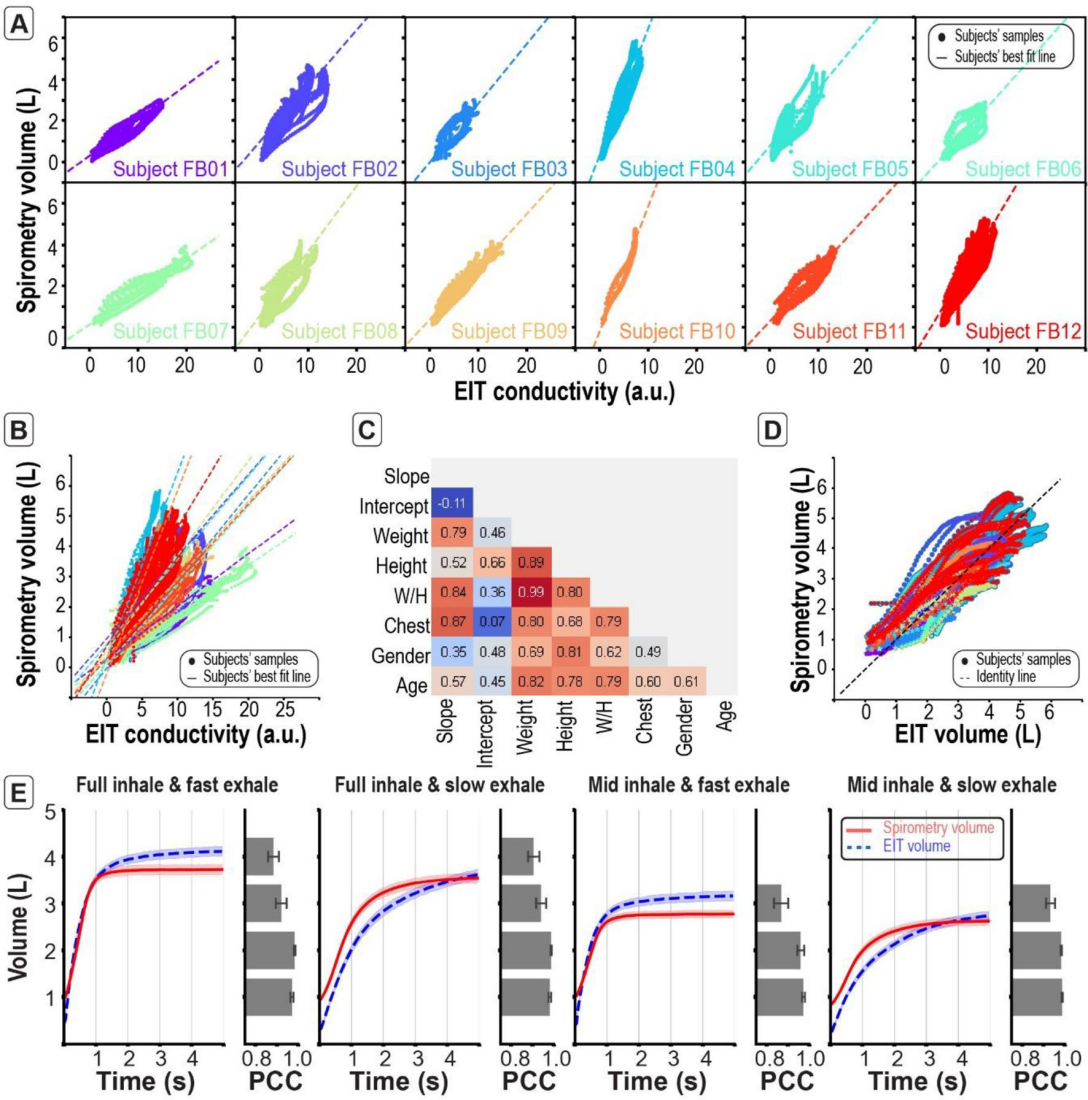
With anthropometric correction, the developed EIT system can be used as a standalone device to predict lung volume change over time. (**A**) Global EIT conductivity-time curves are significantly correlated with volume-time curves for individual subjects. These indicate that the relation between conductivity and volume are quasi-linear and subject-dependent. (**B**) The relation between conductivity-time curves and volume-time curves is subject dependent. (**C**) The correlation matrix between the slope, intercept and subjects’ anthropometrics shows that the slope is highly correlated with the weight-height ratio (W/H) and the chest circumference (Chest). (**D**) The predicted volume-time curves using EIT and subjects’ anthropometrics are highly correlated with the measured volume-time curves. The train and test samples are shown in Supplementary Figure S5 (**E**) The mean (± standard error) of the predicted volume-time curves is in agreement with the mean (± standard error) of the measured volume-time curves for all breathing efforts as shown by the bar plots of the Pearson’s correlation coefficient (PCC; ± standard error) computed on the volume segments 0-1L, 1-2L, 2-3L, and 3-4L.

The scatter plots between the conductivity and volume changes for different subjects are presented in Fig. 2A. The relationship between conductivity and volume is linear with an average PCC equal to 0.89 ± 0.02 (*p* < 0.001) for all subjects. A simple linear regression is performed to obtain the best fit line that predicts the volume from conductivity on different subjects. Note the slopes of the best fit lines are different for each subject (Fig. 2B), implying the volume change cannot be purely explained with conductivity change for different subjects (PCC = 0.52). The correlation between the slope and intercept, and subjects’ anthropometrics such as age, gender, height, weight, weight-height ratio ($\mathrm{W}/\mathrm{H}$) and chest circumference (Chest) are evaluated and shown in Fig. 2C and Supplementary Figure S4, which shows that the slope has a strong correlation with the weight, weight-height ratio and chest circumference (PCC > 0.8; *p* < 0.001). This implies that subjects with larger weight/height and/or chest circumference has larger slope and hence for those subjects a smaller change in conductivity is related to a larger change in volume. Since both weight/height and chest circumference are proportional to the volume of the conductive medium, the larger these quantities, the larger the resistance and the slower the change in conductivity. Hence, the weight/height and the chest circumference are used in addition to the conductivity to train a regression model for predicting the volume. The sought model should have a W/H and chest-dependent slope. This is equivalent to a linear regression model with dependent variables which include product terms of anthropometric parameters and conductivity. Since the W/H ratio is strongly correlated with the weight (PCC > 0.98; *p* < 0.001), weight was not included to avoid multi-collinearity.

To ensure that the regression model is capable of generalizing to unseen data from subjects with different anthropometrics and to fairly evaluate the model performance, the data is split into training- and testing-samples. The test-samples are obtained by randomly excluding all data from two subjects and another 10% of the data from the remaining subjects. The training-samples are the remaining data. Note the proportion of the testing-samples is 24% from the total samples. The predicted volume and the measured volume are significantly correlated (Fig. 2D, Supplementary Figure S5) for both training (PCC = 0.89; *p* < 0.001) and testing-samples (PCC = 0.8; *p* < 0.001). The normalized root mean squared error (NRMSE) of the predicted volume is 10.4% and 13.4% for the training- and testing-samples, respectively. These errors are of the same order of magnitude as in previous studies^16^. This shows that the developed regression model can predict the volume from the conductivity and anthropometrics for a wide dynamic range and for different subjects of different anthropometrics. The predicted volume over time for different effort level agree with the measured volume (Fig. 2E, PCC > 0.8; *p* < 0.001 for all volume segments).

### Spirometry indicators predicted using EIT

**Figure 3:**
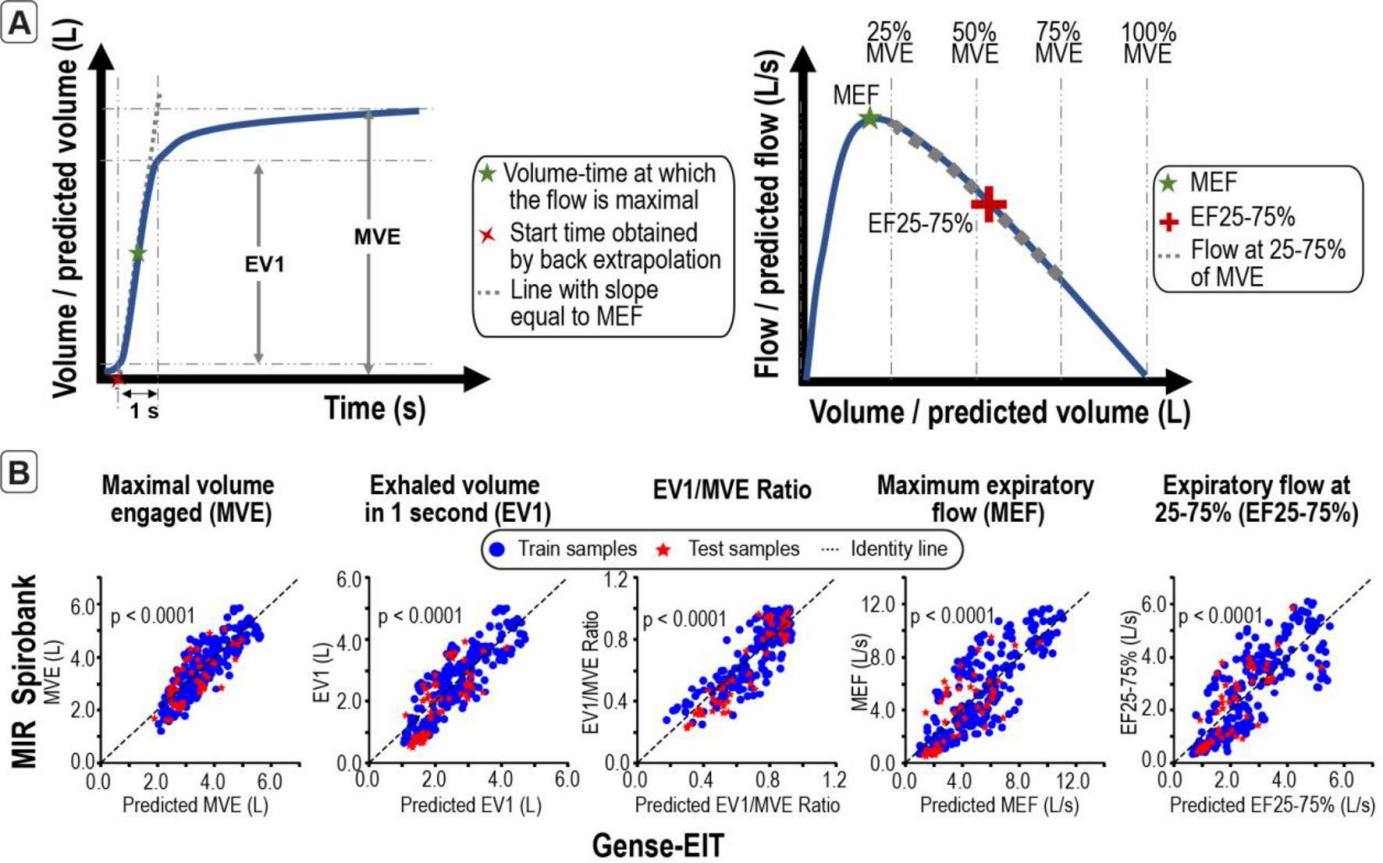
The spirometry indicators predicted with the EIT device are significantly correlated with the indicators from a standard spirometer, demonstrating the capability of the developed EIT system to infer standard lung function indicators. (**A**) Illustration of the method used for calculating the spirometry indicators^7^ (**B**) EIT-derived indicators are significantly correlated with spirometry indicators across a wide dynamic range for both training and testing samples, demonstrating that the device has standard spirometry capabilities. Abbreviations: Maximal volume engaged (MVE), exhaled volume in 1 s (EV1), maximum expiratory flow (MEF), expiratory flow at 25–75% of maximum volume engaged (EF25-75%).

From diagnosis point-of-view, spirometry indicators are considered the gold standard, and is the most widely used diagnostic test for asthma, chronic obstructive pulmonary disease (COPD) and other obstructive or restrictive lung diseases^24^. The most widely used spirometry indicators are forced vital capacity (FVC), forced expiration volume in 1 s (FEV1), FEV1/FVC ratio, peak expiratory flow (PEF) and forced expiratory flow at 25–75% of forced vital capacity (FEF25–75%). It is therefore important to ensure our EIT device can predict these indicators. Since not all customized breathing paradigms involve full-effort forced exhalation, these indicators are referred as maximal volume engaged (MVE), exhaled volume in 1 s (EV1), EV1/MVE ratio, maximum expiratory flow (MEF), and expiratory flow at 25–75% of maximum volume engaged (EF25-75%), corresponding to FVC, FEV1, FEV1/FVC, PEF, FEF25-75% respectively. These EIT-derived indicators are calculated as follows (Fig. 3A). MVE is obtained by the difference between the maximum and minimum predicted volume changes; EV1 is the change in predicted volume within the first second from the onset of exhalation; EV1/MVE is the ratio of EV1 and MVE; MEF is the maximum value of the time derivative of the predicted volume curve; and EF25-75% is the average of the time derivative of the predicted volume during the expiration from 25% of MVE to 75% of MVE. The scatter plots in Fig. 3B show the spirometry indicators predicted from our EIT device versus the measured spirometry indicators. All indicators predicted using our device are significantly correlated with the spirometry indicators (PCC > 0.7; *p* < 0.001; online methods, Supplementary Table S2).

### Regional spirometry indicators are consistent with corresponding breathing paradigms

**Figure 4:**
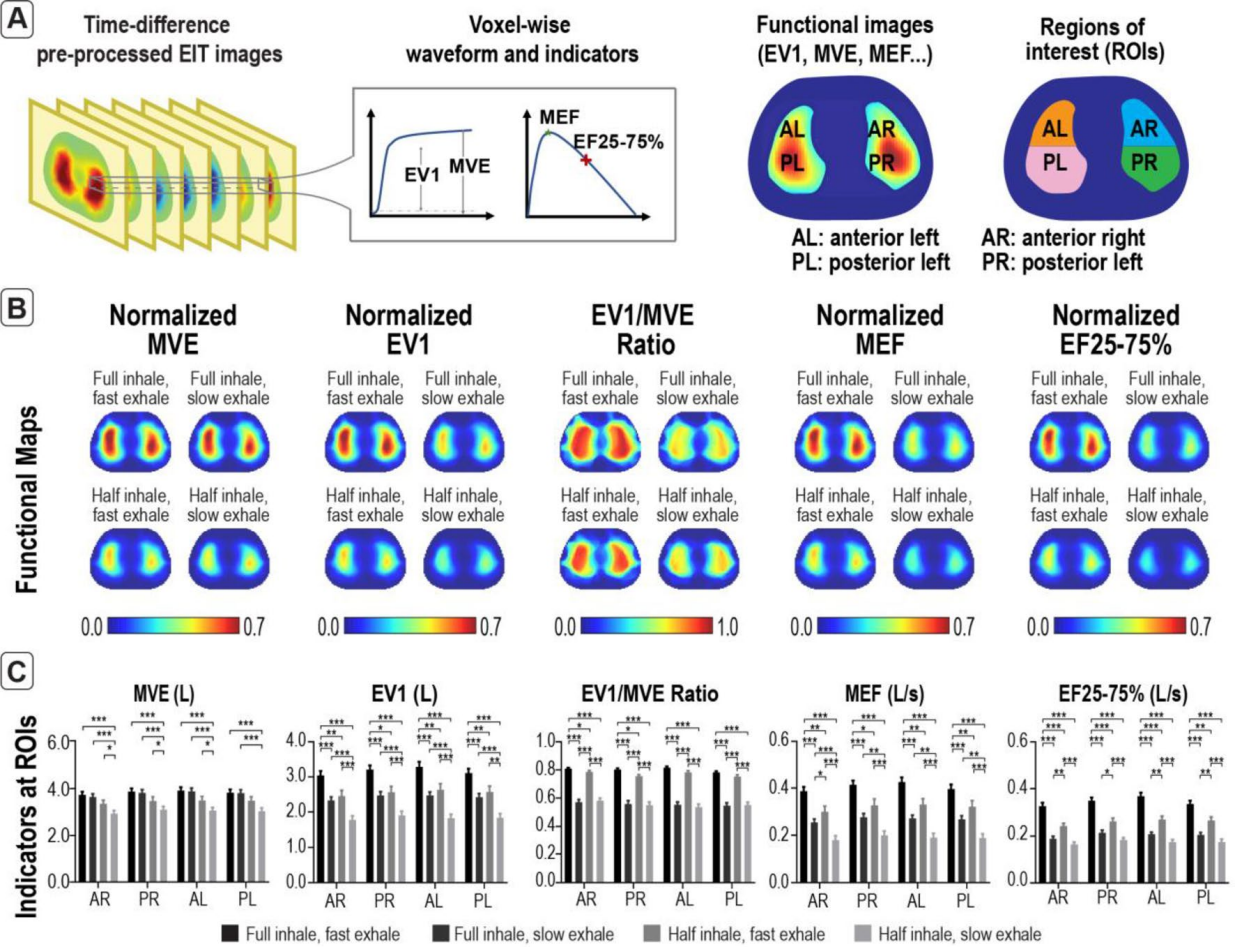
EIT-derived functional maps and regional EIT-derived indicators were consistent with the four corresponding breathing paradigms which is the combination of full or mid capacity inhale and fast or slow exhale. (**A**) Functional maps are obtained by evaluating the EIT indicators from the voxel-wise waveforms from which the regions of interest (ROIs) are inferred, including anterior left (AL), posterior left (PL), anterior right (AR) and posterior right (PR). (**B**) Averaged functional maps were calculated, allowing regional lung function assessment. (**C**) All regional indicators followed a similar trend, i.e., the MVE is higher in paradigms involving full capacity inhale, the EV1 is highest in full capacity inhale with fast exhale and is lowest in half capacity inhale with slow exhale, the EV1/MVE ratio is higher in paradigms involving fast exhale, the MEF is highest in full capacity inhale with fast exhale and is lowest in half capacity inhale with slow exhale, and the EF25-75% is highest in full capacity inhale with fast exhale and is lowest in slow exhale. *p < 0.05, **p < 0.01, ***p < 0.001. Error bars indicate ± standard error of mean. Abbreviations: Maximal volume engaged (MVE), exhaled volume in 1 s (EV1), maximum expiratory flow (MEF), expiratory flow at 25–75% of maximum volume engaged (EF25-75%).

The same indicators MVE, EV1, EV1/MVE, MEF and EF25-75% are also computed at a voxel-level (functional maps). Indicators at voxels having a low correlation with the global waveform and small amplitude are set to zero to reduce the noise in the functional maps (Fig. 4A). The left and right lung clusters are further partitioned into posterior and anterior partitions (i.e., anterior left (AL), posterior left (PL), anterior right (AR) and posterior right (PR)) in which the indicators are evaluated. The averaged functional maps of each EIT-derived indicator from all the breathing paradigms are shown in Fig. 4B. All EIT-derived indicators extracted from four ROIs (Fig. 4C) shared a similar trend as the global, i.e., the MVE is higher in paradigms involving full capacity inhale (*p* < 0.001), the EV1 is highest in full capacity inhale with fast exhale (*p* < 0.001) and is lowest in half capacity inhale with slow exhale (*p* < 0.001), the EV1/MVE ratio is higher in paradigms involving fast exhale (*p* < 0.001), the MEF is highest in full capacity inhale with fast exhale (*p* < 0.001) and is lowest in half capacity inhale with slow exhale (*p* < 0.001), and the EF25-75% is highest in full capacity inhale with fast exhale (*p* < 0.001) and is lowest in slow exhale (*p* < 0.001).

### Novel close-to-effortless guided breathing paradigm

**Figure 5:**
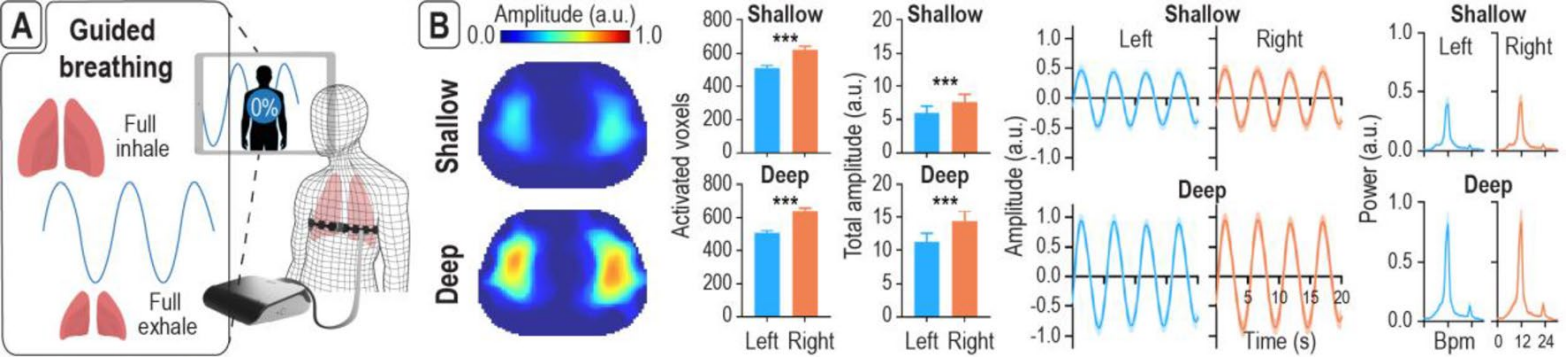
EIT with novel close-to-effortless guided breathing paradigm can quantify global and regional lung functional changes. (**A**) The paradigm is a periodic inhale and exhale pattern at 12 breaths per minute (bpm). (**B**) Shallow and deep breathing modes were applied to assess the sensitivity of this method. Activated voxels and total amplitude were extracted from the left and right lungs. The right lung has significantly more activated voxels and significantly higher total amplitude compared to the left. The amplitude maps, total amplitude, conductivity time curve, and frequency spectra exhibited higher amplitude during deep breathing compared to shallow breathing, while activated voxels remained similar. ***p < 0.001. Error bars indicate ± standard error of mean. Abbreviations: arbitrary unit (a.u.). The drawings of the electrode belt and the portable EIT console were made using Dassault Systemes Solidworks 2020, Luxion Keyshot 9 and Adobe Photoshop CC 2019. The drawings of the dummy person was made using Adobe Illustrator 2021.

We further developed a novel guided breathing paradigm which consists of a periodic inhalation and exhalation at 12 breaths per minute (bpm) (Fig. 5A), and its corresponding processing pipeline (see online methods). The choice of a relatively slow breathing rate (typical range: 12–20 bpm^25^) is to impose a certain degree of breathing challenge. That is a normal subject would tend to breathe a larger amount of air at each cycle during this exercise. A total of nine subjects of different age, height, weight, ethnicity and predicted FVC performed more than four repetitions of guided breathing (demographic and anthropometric details are in Supplementary Table S3). Subjects were instructed to perform both deep and shallow breathing modes to characterize the functional differences. From each subject and each repetition, we computed the amplitude maps, total amplitude, conductivity-time curve, and frequency spectra (see online methods). All the results were normalized by the largest subject-dependent participant to strictly compare the two breathing modes (see online methods). As expected, the deep breathing mode exhibited higher amplitude as compared to shallow breathing (*p* < 0.001, Fig. 5B and Supplementary Figure S6). Note the activated voxels are nearly the same for both breathing depths, the total amplitude was higher during deep breathing (*p* < 0.001). However, the right lung has significantly more activated voxels (*p* < 0.001) and significantly higher total amplitude (*p* < 0.001) compared to the left in both shallow and deep breathing modes, likely due to the positioning of the heart within the left thorax. The normalized average conductivity in the right and left lung lobes are shown in Fig. 5B both in the time and frequency domains. The results show that the conductivity variations for both lungs and breathing modes follow a periodic oscillation of twelve periods per minute with lower amplitude for shallow breathing. These results demonstrated that this novel close-to-effortless guided breathing paradigm can reflect both global and regional lung function changes.

### COVID-19 case study

**Figure 6:**
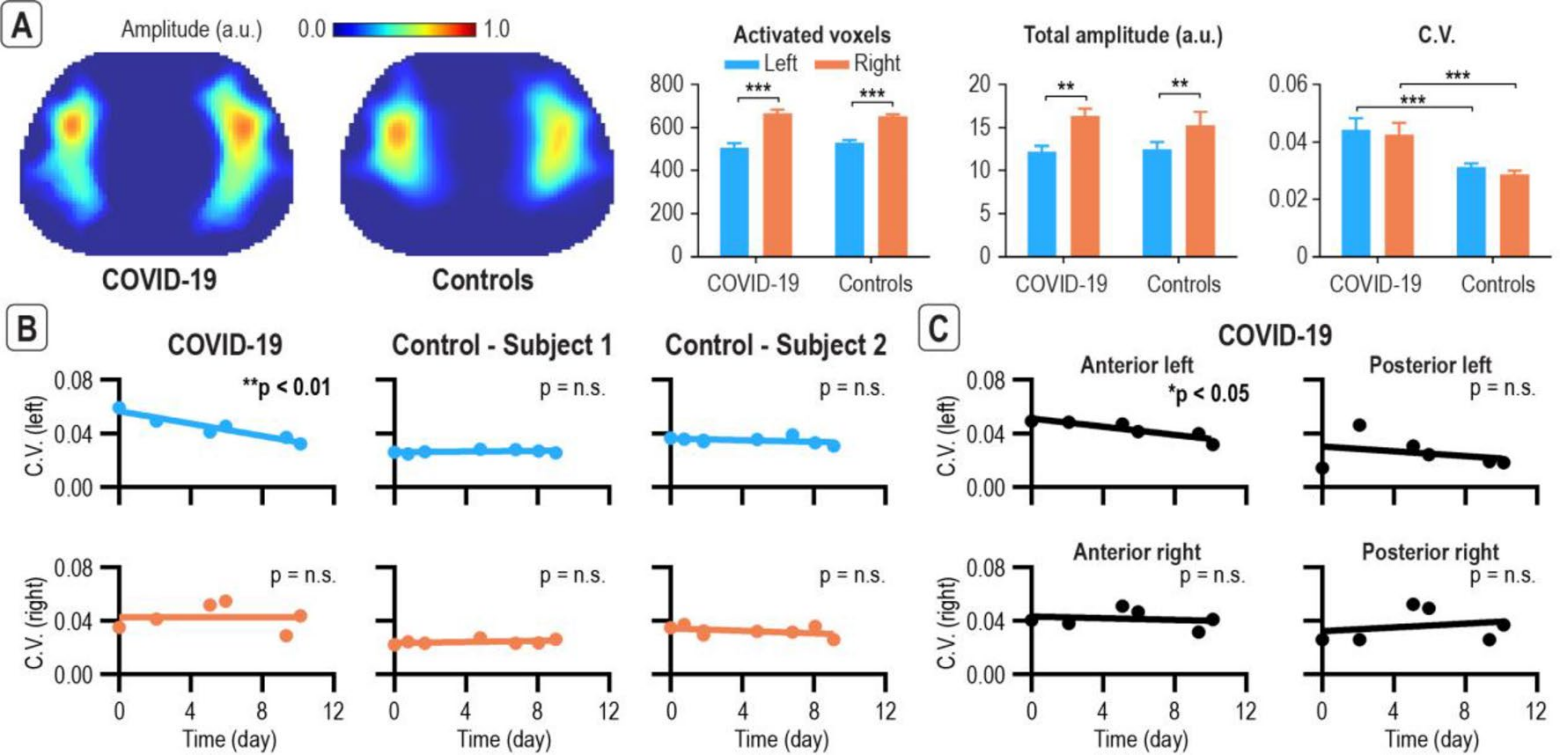
EIT with novel guided breathing paradigm close-to-effortless can detect regional lung deterioration followed by a recovery for a COVID-19 discharged subject. COVID-19 discharged subject was longitudinally monitored along with two age- and gender-matched controls (**A**) The COVID-19 discharged subject had higher C.V., suggesting lung function deterioration. Note the right lung has significantly more activated voxels and significantly higher total amplitude. (**B**) C.V. significantly decreased across time in the left lung of the COVID-19 discharged subject, suggesting a functional deterioration at the beginning followed by a recovery. (**C**) Regional analysis further pin-pointed deterioration and recovery in the anterior left lung. *p < 0.05, **p < 0.01, and ***p < 0.001. Error bars indicate ± standard error of mean. Abbreviations: arbitrary unit (a.u.); non-significant (n.s.); coefficient of variation (C.V.).

To further characterize the system performance with this novel close-to-effortless paradigm, a COVID-19 discharged subject was longitudinally monitored with two healthy controls. The COVID subject and the two healthy controls were selected such that they are all non-smokers, have no known history of heart and/or lung diseases such as COPD, Asthma, non-treated Bronchitis or pneumonia, have the same gender (all male), similar predicted FVC (5.6L (COVID), 5.3L, 5.2L), age (33 (COVID), 24, 28), weight (79 kg (COVID), 75 kg, 58 kg), and chest circumference (90 cm (COVID), 89 cm, 81 cm) (demographic, anthropometric and clinical details in Supplementary Table S4). Both COVID discharged subject and the two healthy controls performed the tests while standing with the lung belt around the T4 and T5 vertebrae and were instructed to follow the breathing paradigm on the screen (Fig. 5A) with no specific instruction on breathing depth. The averaged amplitude map across different trials for the COVID-19 subject and the two controls are shown in Fig. 6A together with their associated indicators including activated voxels, total amplitude, and coefficient of variation (C.V., see online methods). The coefficient of variation is an indicator of the degree of inhomogeneity at different lung regions and was shown to be indicative of lung abnormality in previous studies^26–28^. The activated voxels and total amplitude for all subjects are consistent with the results from the guided breathing experiment with variable depth, i.e., the right lung has significantly more activated voxels and significantly higher total amplitude (*p* < 0.001). On the other hand, the COVID-19 discharged subject had higher C.V. (*p* < 0.001), suggesting lung function deterioration.

To further examine the regional lung functions, time evolutions of the C.V. of the left and right lungs are presented in scatter plots and linearly regressed (Fig. 6B). The results suggest that the C.V. significantly decreased (*p* < 0.01) across time in the left lung of the COVID-19 discharged subject (Fig. 6B), suggesting a functional deterioration at the beginning followed by a recovery. Whereas no significant trends were observed for the controls. The time evolution of the C.V. is further evaluated at the anterior and posterior of the left and right ROIs. The results suggest that the potential deterioration and recovery has specifically occurred in the anterior left lung (Fig. 6C). Given that this is a single-subject case study, more extensive tests are required to support these conclusions.

## Discussion

Here, we designed and developed a home-based self-administrable, portable, and cost-effective EIT system, implemented a close-to-effortless breathing paradigm, and demonstrated its feasibility to assess global and regional lung functions longitudinally.

### EIT has standard spirometry capabilities with additional spatial information

We validated the developed portable EIT system as a standalone device to predict standard spirometry indicators. Our results demonstrated that the volume of airflow through the lung can be predicted using EIT and subject’s anthropometrics without calibration (PCC > 0.8; *p* < 0.001). We observed a slight difference between EIT conductivity-time curve and spirometry volume-time curve, potentially due to the fact that the EIT imaging slice cannot represent the whole lung (Supplementary Figure S7), which may be elucidated with 3D-EIT^10^ in the future. We also showed that EIT can infer standard spirometry indicators (PCC > 0.7; *p* < 0.001), making it potentially suitable for screening, diagnosis, and monitoring of obstructive and restrictive lung diseases, facilitating its adoption as a standard screening tool for lung function assessment. In addition, we showed that the system provides regional functional mapping of spirometry indicators. We hypothesize that such additional spatial information would be critical for regional lung functional assessment, which can be essential for detecting and monitoring regional changes in lung diseases, such as COPD. Future clinical studies involving diseased patients such as COPD, asthma, and interstitial lung disease are warranted.

### Uncertainty of the predicted spirometry indicators due to the variability of the belt position, subject posture, and repetition

To assess the uncertainties of the device due to the variability of the belt position, subject posture, and repetition, we repeatedly acquired simultaneous EIT and spirometry measurement from two subjects during forced breathing of different efforts. The first subject performed the tests while varying the electrodes belt position and his posture. The belt position variability test is conducted while the subject is standing and the belt is placed at four different positions: (i) around the T4 and T5 vertebrae (instructed or “normal” position), (ii) 1 cm above (i.e., around T4), (iii) 2 cm below (i.e., between T5 and T6) and (iv) 5 cm below (i.e., between T7 and T8)^29^. The posture variability test was conducted while the belt is placed at the instructed position and the subject is holding three different postures: (i) standing (instructed or “normal” position), (ii) sitting, (iii) laying. The variability due to the repetition test is conducted by the second subject on two different days (day 1 and day 11) while standing with the lung belt around the T4 and T5 vertebrae. The scatter plot comparing the EIT indicators, and the spirometry indicators are shown in Supplementary Figures S8, S9 and S10. The data acquired from the instructed position or day 1 is used to create a regression model to predict the spirometry indicators from the EIT indicators. The regression model is then applied to the data acquired at other belt positions or day. The NRMSE induced by varying the belt 1 cm above to 5 cm below are comparable to the ones obtained from the normal position, with the largest errors recorded when the belt is 5 cm the instructed position (Supplementary Table S3). The NRMSE induced by varying the subject’s posture from standing, sitting to laying are comparable with the largest errors recorded when the subject is laying (Supplementary Table S3). Finally, the NRMSE induced by repeating the tests on day 11 are comparable to the errors at day 1, with the errors at day 11 larger (Supplementary Table S3). Although, the results suggest that the prediction of the spirometry indicators is not greatly affected by small errors in the belt position, subject posture, and repetition, it should be noted that the errors for these experiments were evaluated on a single subject. Hence, further experiments are needed to accurately estimate the error induced by these variabilities. Finally, it should be noted that compensating for the errors induced by the belt position or subject posture is also possible^16^. The development of such a model requires data to be acquired from multiple subjects, which will be considered for future studies.

### Monitor COVID-19 recovery and other chronic pulmonary diseases

The novel close-to-effortless guided breathing paradigm is sensitive to different breathing efforts and provides both global and regional lung function evaluations. Furthermore, the longitudinal monitoring of the COVID-19 discharged subject showed that the EIT system can potentially detect initial deterioration followed by recovery. While, the recruited subject did not undergo ventilation inhomogeneity (VIH) test or CT-scan to compare our results to these standard test, other studies suggest that inhomogeneity is detected in more than 40% of COVID-19 subjects^30^. More extensive tests with COVID-19 subjects are required to support these conclusions given this is a case study only. The effectiveness of the guided breathing paradigm is yet to be elucidated through extensive clinical trials. Nevertheless, our device can potentially be useful for longitudinal monitoring of COVID-19 recovery. In fact, a complete recovery from COVID-19 may take as long as several months^31,32^. Long COVID is a term devised by patients to describe the lingering symptoms they experience well after an initial bout of COVID-19, with symptoms including shortness of breath, lung structural damage, abnormal lung function, etc. Several studies showed that more than 70% of discharged patients, with two negative tests issued 24 h apart, were found with abnormalities by high-resolution computed tomography (HRCT) scans and spirometry^33,34^. Therefore, the post-treatment monitoring of COVID and longitudinal monitoring of long COVID is desirable for capturing any missed or hidden lung function abnormalities as to improve patient outcomes. Moreover, several respiratory diseases can also be explored by using our EIT approach, including COPD^28,35^, asthma^36^ and acute lower respiratory tract infections^37^.

### Affordable, portable, and self-administrable EIT enables its deployment for telemedicine applications

One of the major advantages of our developed EIT system is its high accessibility which is critical for promoting remote healthcare. Recently, during COVID-19 pandemic, telehealth and telemedicine applications are increasingly demanded worldwide^38^. Telemedicine applications refers to the uses of telecommunication and information technology to provide remote access to health assessment, diagnosis, intervention and consultation, as to assist distal clinical consultation and treatment supervision^39^. In addition to improving access to healthcare, our EIT console and silicon belt serve as self-administrable portable wearable with detailed guidance via mobile app and real time cloud-based data processing and storage, enabling remote monitoring for medical professionals to virtually evaluate patients’ health and treatment responses. Compared to traditional EIT system, our portable EIT system is designed as an affordable telemedicine gadget, which can be widely used for remote patients monitoring under both home-based and clinical settings. It is important to note that a large portion of the cost of the EIT systems may be due to the business decisions and medical licensing. By making the device affordable, portable and self-administrable, larger number of devices can be sold and hence the cost of medical licensing per unit device can be reduced. Ultimately, the proposed device can improve the quality of healthcare of patients suffering from chronic pulmonary diseases^40,41^, reduce both time and cost associated with on-site monitoring^4^, and potentially reduce the overall mortality rates.

### Monitoring chronic diseases of different body parts

Besides pulmonary diseases screening and monitoring, the developed portable EIT system also has the potential for home-based diagnostic screening and treatment monitoring of chronic diseases at other body parts such as the liver, kidney, breast, and synovial joints. In fact, the dielectric properties (e.g., bioimpedance, conductivity, permittivity) could be varied between healthy and diseased tissues at different excitation frequencies^42^. For instance, the conductivity of human ex vivo liver tumor tissues were shown to be higher^43^, while lower conductivity^44^ and higher bioimpedance^45^ were detected from liver steatosis tissue on rats in vivo when compared to healthy liver tissue. Also, bioimpedance of human renal tumor tissues could be lower when current was applied between 200 kHz and 1 MHz in two recent ex vivo kidney studies^46,47^. Besides, ex vivo human breast cancer tissues were shown to have lower bioimpedance^48^, while in vivo human breast cancer tissues had higher conductivity and permittivity^49^. Synovial joints, such as wrists and knees, showed lower bioimpedance in carpal tunnel syndrome patients^50^ and higher bioimpedance in patients with osteoarthritis^51^, respectively. Overall, we expect our portable EIT system to be widely applicable to home-based diagnostic screening and remote treatment monitoring of multiple chronic diseases at different body parts.

## Supplementary Information

Supplementary Information 1.

Supplementary Information 2.

Supplementary Information 3.

Supplementary Information 4.

Supplementary Information 5.

## Data Availability

Representative datasets (including raw data, interim steps and final results) and the codes supporting the findings in this manuscript are available at https://drive.google.com/drive/folders/1eOxVvpp66FQmWZTWLYzmVsLdV2zjI_EI?usp=sharing upon published. Full datasets are available from the corresponding author upon reasonable request.
