# Supplementary material for: Affordable, portable and self-administrable electrical impedance tomography enables global and regional lung function assessment
Source: Sci Rep. 2022 Nov 30;12:20613. doi: 10.1038/s41598-022-24330-2 (PMC9712422; doi:10.1038/s41598-022-24330-2)
Supplement: Supplementary file 1 — Supplementary Information 1. [file 41598_2022_24330_MOESM1_ESM.docx]

**Supplementary Information**

**Affordable, portable and self-administrable electrical impedance tomography enables global and regional lung function assessment**

Fedi Zouari^1^, Wei Yi Oon^1,5^, Dipyaman Modak^1^, Wing Hang Lee^1^, Wang Chun Kwok^2,3^, Peng Cao^4^,
Wei-Ning Lee^5^, Terence Chi Chun Tam^2,3^, Eddie C. Wong^1^, Russell W. Chan^1,*^

^1^Gense Technologies Ltd., Hong Kong, China

^2^Department of Medicine, Queen Mary Hospital, Hong Kong, China

^3^Department of Medicine, Li Ka Shing Faculty of Medicine, The University of Hong Kong, Hong Kong, China

^4^Department of Diagnostic Radiology, Li Ka Shing Faculty of Medicine, The University of Hong Kong, Hong Kong, China

^5^Department of Electrical and Electronic Engineering, Faculty of Engineering, The University of Hong Kong, Hong Kong, China

*Correspondence to:

Russell W. Chan, Ph.D.

[russell@gense.tech](mailto:russell@gense.tech)

# Supplementary notes

**Supplementary note 1**

Subjects’ exclusion criteria

- Subjects with implanted electronic devices, in case EIT might interfere with the devices
- Subjects with spinal diseases/ discomfort
- Subjects with unstable heart/ lung conditions
- Pregnant women
- Patients who are unable to perform spirometry (Unable to follow instructions, have end-tracheostomy etc.)

# Supplementary figures and captions


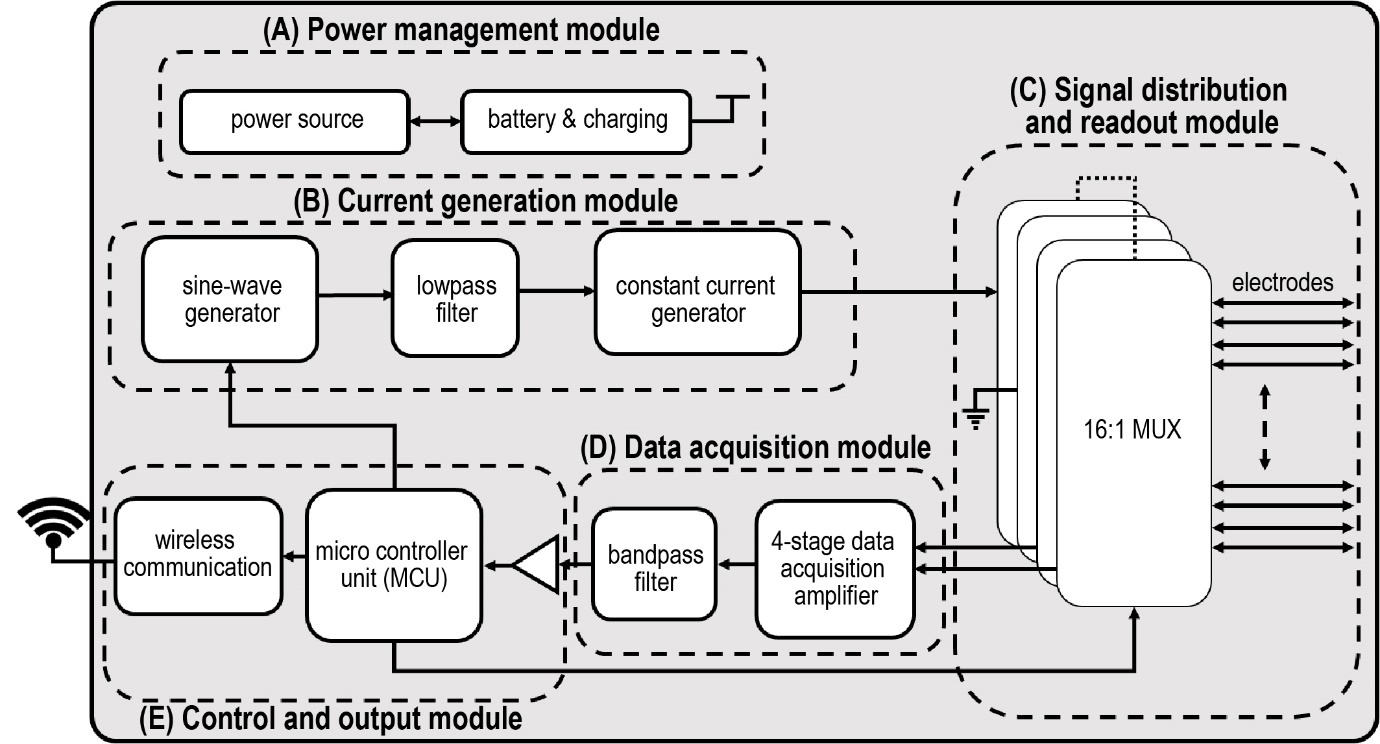


**Supplementary Figure S1: The** **portable EIT console consists of 5 modules. (A)** **Power management module** provides constant power supply to all other modules through the power socket or the Li-ion battery. **(B)** **Current generation module** primarily includes a digitally programmable analog sine wave generator and a constant current generator successively to generate an alternating current of 1 mApp and a voltage amplitude of 1 Vpp. A low-pass filter is used to suppress total harmonic distortion and ambient electromagnetic interference (e.g., power line noise). **(C)** **Signal distribution and readout module** introduces the generated current to the subject via the 16-electrode belt using a set of CMOS multiplexers (MUXs). Four MUXs are used, in which two MUXs are employed for current injection and the other two for voltage readout. The MUXs are configured into the adjacent-scan pattern through the microcontroller unit (MCU). **(D)** **Data acquisition module** is the analog front-end (AFE) that acquires, measures and amplifies the potential differences from the electrodes. The AFE comprises a four-stage wide input differential amplifier with high common-mode rejection ratio (CMRR), and a bandpass filter. **(E)** **Control and output module** consists of an analog-to-digital converter (ADC), a MCU and a wireless communication chip. The potential differences obtained from the data acquisition module are digitized by a 12-bit ADC, processed in the MCU unit, and transferred to the cloud server for image reconstruction and processing.


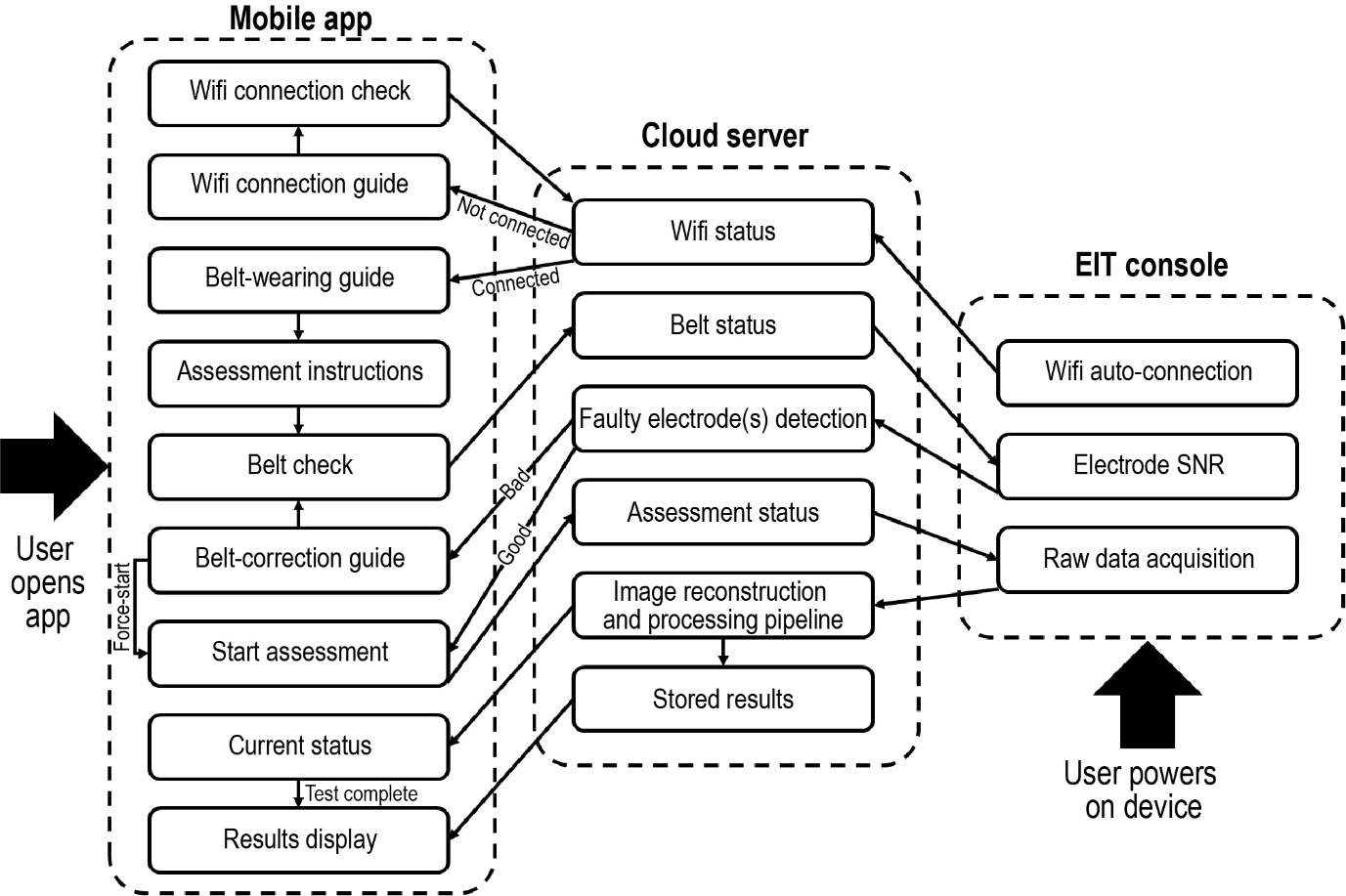


**Supplementary Figure S2: Lung function assessment is achieved through the coordination between the mobile app, cloud server, and EIT console.** The EIT console is first connected to wifi, either automatically or manually. Users will then be instructed to wear the electrode belt, and electrode quality will be analyzed. Once a high signal-to-noise ratio (SNR) is achieved or a force-start is triggered, the lung function assessment will begin. Finally, the acquired raw data will be transferred to the image reconstruction and processing pipeline, and results will be displayed.


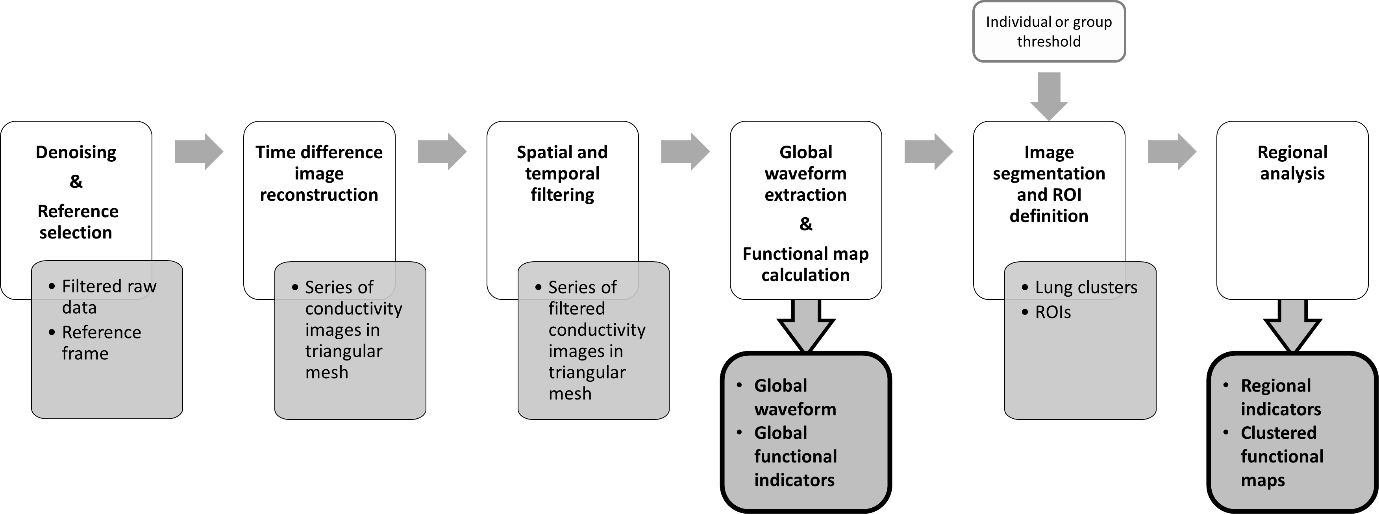


**Supplementary Figure S3: Global and regional indicators and functional maps are obtained through multiple processing stages.** A reference is first chosen from the measured voltage data then the raw data is denoised. Time difference conductivity images are then reconstructed from all measured frames and from the reference data. Subsequently, spatial and temporal filtering is applied to the series of reconstructed images from which global functional indicators and waveform are extracted. The series of EIT images and the global waveform are used to define the regions of interest which are then used to extract regional indicators and functional maps.


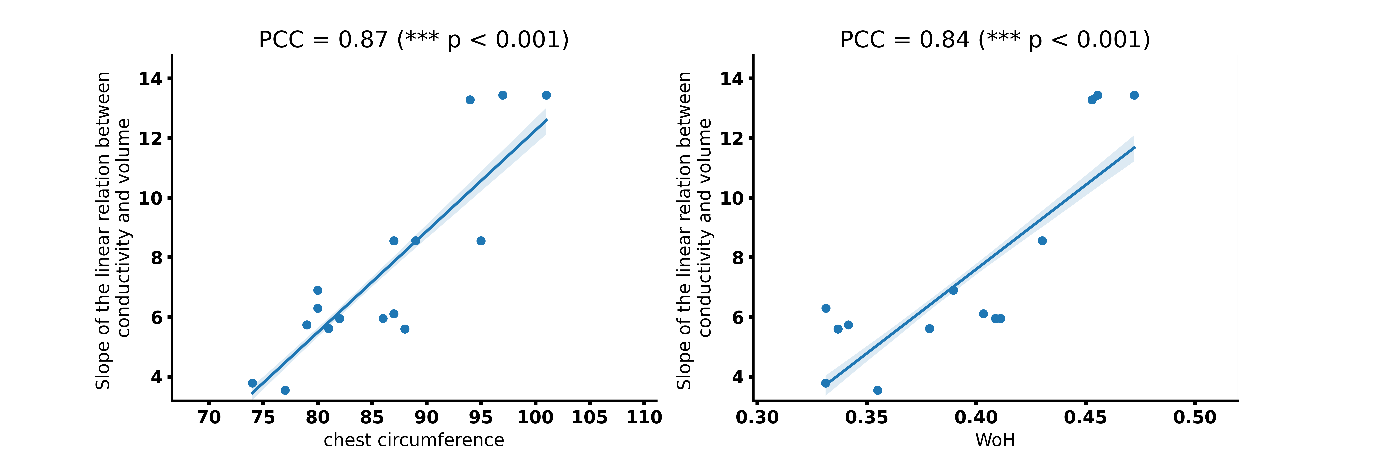


**Supplementary Figure S4: The slope of the predicted subject-wise linear relation between conductivity and volume using our EIT device is significantly correlated with the subject’s chest circumference and weight over height ratio (WoH).** (Left) The slope of the linear relation between conductivity and volume for different subjects is significantly correlated to the chest circumference with a Pearson’s correlation coefficient (PCC) of 0.87 (p<0.001). (Right) The slope of the linear relation between conductivity and volume for different subjects is significantly correlated to the weight over height ratio (WoH) with PCC= 0.84 (p<0.001).


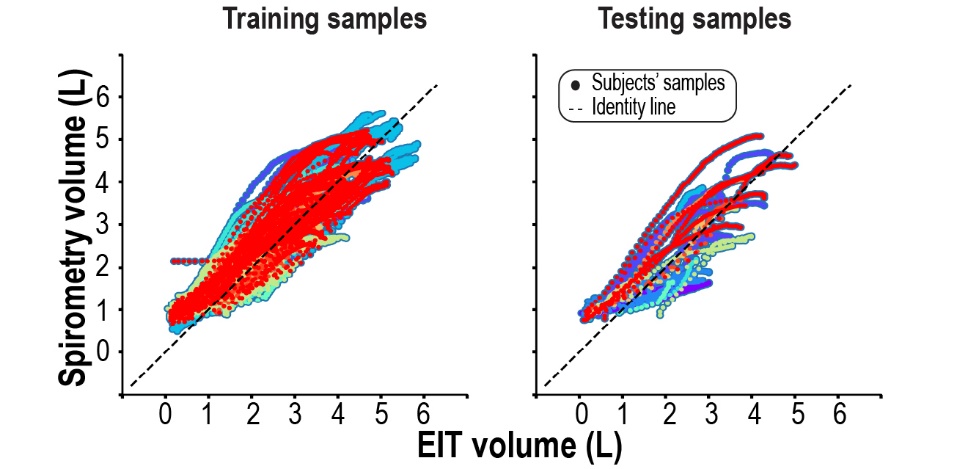


**Supplementary Figure S5: The predicted volume using our EIT device is significantly correlated with the measured volume using spirometry.** (Right) Training samples have a Pearson’s correlation coefficient (PCC) of 0.89 (p<0.001) and a normalized root mean squared error (NRMSE) of 10%. (Left) Testing samples have PCC=0.8 and NRMSE=13.4%.


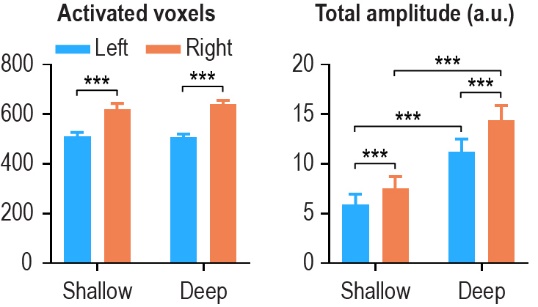


**Supplementary Figure S6: EIT with novel guided breathing paradigm close-to-effortless can quantify lung functional changes.** Shallow and deep breathing modes were applied to test the sensitivity of this close-to-effortless breathing paradigm. The right lung has significantly more activated voxels and significantly higher total amplitude compared to the left in both shallow and deep breathing modes, likely due to the positioning of the heart within the left thorax. The total amplitude was higher during deep breathing compared to shallow breathing, while activated voxels remained similar. ***p < 0.001. Error bars indicate ± standard error of mean. Abbreviations: arbitrary unit (a.u.).


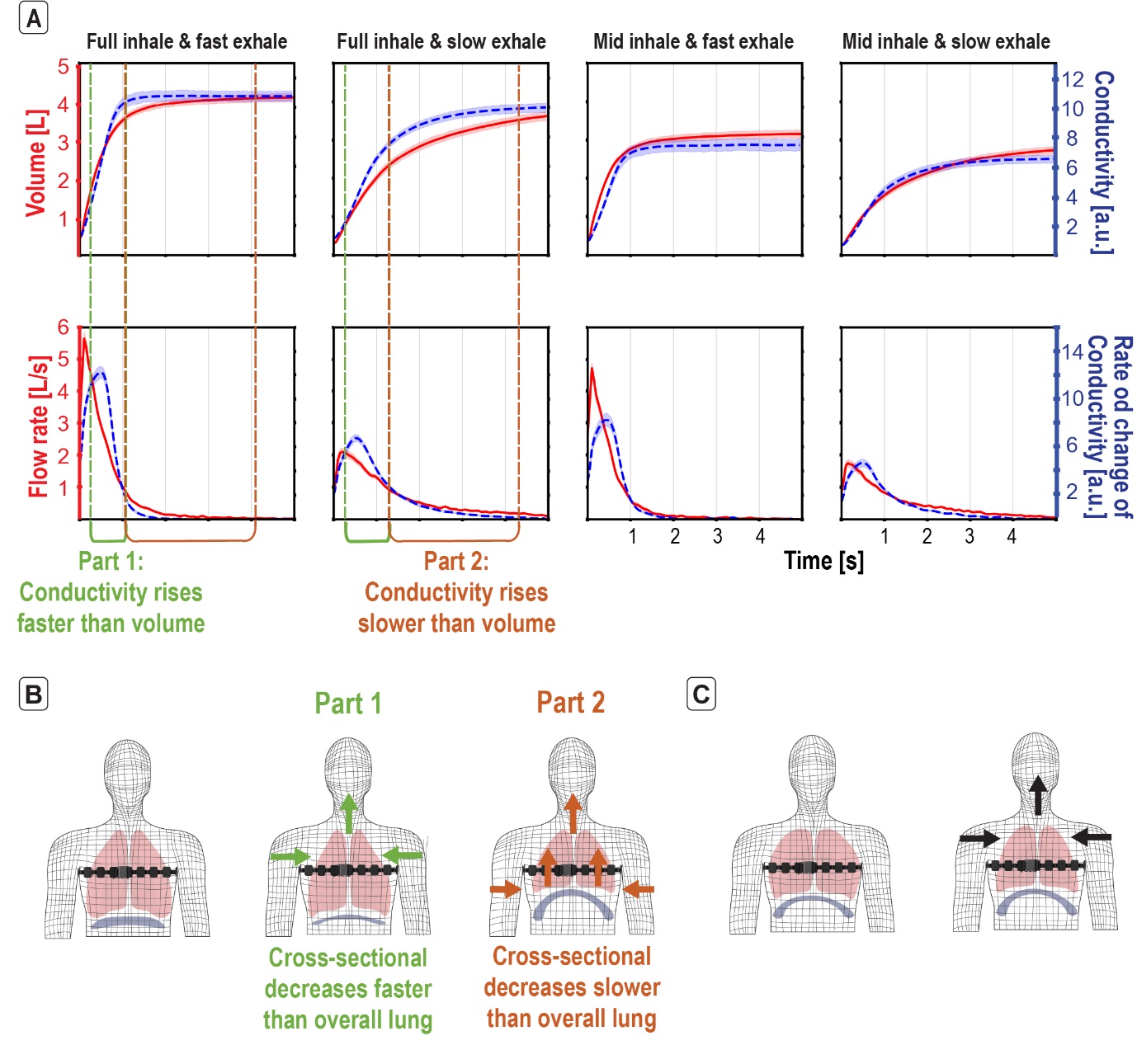


**Supplementary Figure S7: Slight difference between the EIT conductivity-time curve and spirometry volume-time curve was observed. (A)** We observed two distinct regimes for paradigms involving full inhale. Part 1, the conductivity-time curve is increasing more rapidly compared to the volume-time curve. Part 2, the conductivity-time curve is increasing at a much slower rate as compared to the volume-time curve. **(B)** We speculate that the air flows from the upper lung region during Part 1, which is the region captured by EIT (second column). In contrary, the air flows from the lower lung region afterwards (part 2), which is a region different from the one being captured by EIT. During this time, the upper lung region (captured by EIT, i.e., conductivity) does not undergo rapid changes as compared to the overall lung (i.e., volume; third column). **(C)** Half inhale paradigms may engage more of the upper lung than the lower lung, thus, the aforementioned phenomena was not observed.


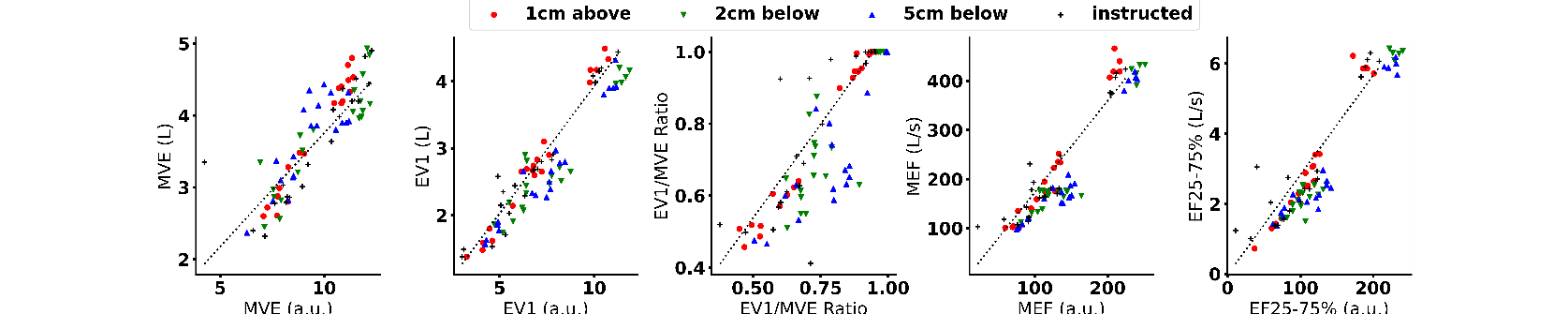


**Supplementary Figure S8: Spirometry indicators compared to EIT indicators during forced breathing while the electrode belt is placed at different positions.** The belt positions considered are the T4 and T5 vertebrae (instructed or “normal” position), 1 cm above, 2cm below and 5 cm below the instructed position. The dotted line represents the best fit line for the data acquired at the instructed belt position.


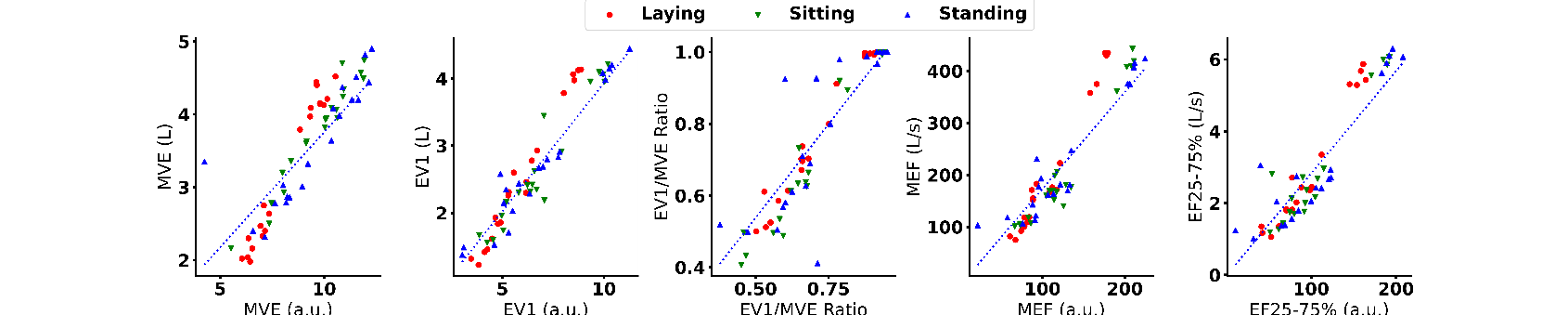


**Supplementary Figure S9: Spirometry indicators compared to EIT indicators during forced breathing while the subject is holding different postures.** The postures considered are the standing (instructed or “normal” posture), sitting, and laying. The dotted line represents the best fit line for the data acquired at the instructed posture.


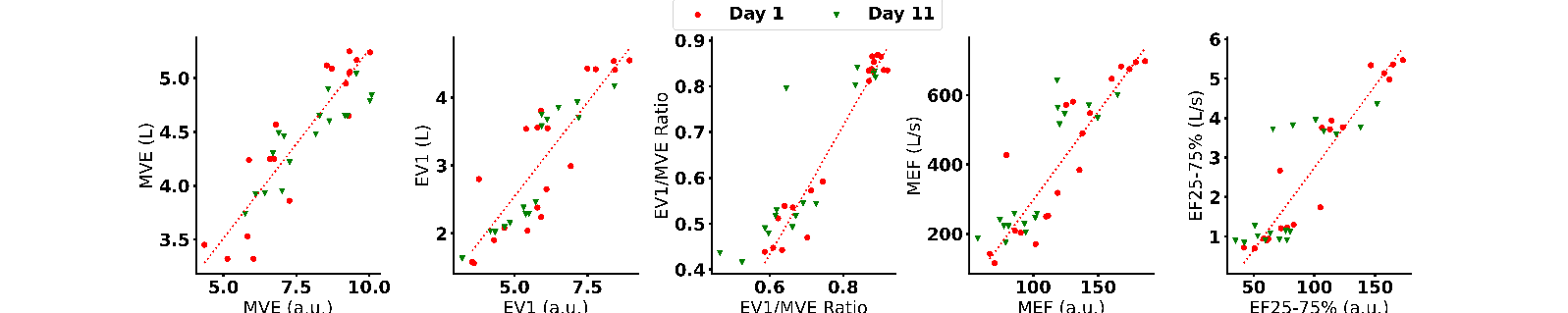


**Supplementary Figure S10: Spirometry indicators compared to EIT indicators during forced breathing for the same subject on different days.** The same subject has performed forced breathing test at two different days: Day 1 and Day 11. The dotted line represents the best fit line for the data acquired at Day 1.

# Supplementary tables and captions

**Supplementary** **Table S1: Anthropometric and demographic information of all 14 subjects who underwent simultaneous EIT and spirometry with four different breathing paradigms in Figure 1.**

| ***Subject*** | ***Gender*** | ***Age*** | ***Place of Origin*** | ***Weight (kg)*** | ***Height (cm)*** | ***BMI (kg/m^2^)*** | ***Chest circumference (cm)*** | ***Predicted FVC (L)*** |
| --- | --- | --- | --- | --- | --- | --- | --- | --- |
| FBDE-01 | Male | 23 | Pakistan | 67 | 172 | 22.65 | 80 | 4.92 |
| FBDE-02 | Female | 19 | Indonesia | 55 | 161 | 21.22 | 75 | 3.57 |
| FBDE-03 | Female | 24 | Hong Kong | 75 | 158 | 30.04 | 90 | 3.42 |
| FBDE-04 | Male | 31 | Hong Kong | 73 | 181 | 22.28 | 87 | 5.35 |
| FBDE-05 | Female | 18 | Indonesia | 51 | 154 | 21.50 | 74 | 3.26 |
| FBDE-06 | Female | 18 | Indonesia | 54 | 163 | 20.32 | 80 | 3.67 |
| FBDE-07 | Male | 21 | Hong Kong | 60 | 169 | 21.01 | 77 | 4.77 |
| FBDE-08 | Female | 21 | Chinese | 53 | 156 | 21.78 | 70.5 | 3.34 |
| FBDE-09 | Male | 24 | Hong Kong | 64 | 169 | 22.41 | 81 | 4.72 |
| FBDE-10 | Male | 30 | Tunisia | 82 | 180 | 25.31 | 101 | 5.3 |
| FBDE-11 | Male | 24 | Malaysia | 77 | 179 | 24.03 | 87 | 5.34 |
| FBDE-12 | Male | 30 | Hong Kong | 74 | 182 | 22.34 | 86 | 5.43 |
| FBDE-13 | Male | 32 | Hong Kong | 77 | 170 | 26.64 | 94 | 4.64 |
| FBDE-14 | Male | 20 | India | 57.6 | 171 | 19.70 | 88 | 4.9 |

**Supplementary Table S2: Evaluation of the regression model used to predict the spirometry indicators.** The test-set is obtained from the global set by randomly excluding all data from two subjects and another 10% of the data from the remaining subjects. The train-set is the remaining data from the global set after excluding the test-set. The performance of the regression model is evaluated with the Pearson’s correlation coefficient (**PCC**) and normalized root mean squared error percentage (**NRMSE%**). The results show that the performance on the train-set and test-set are very similar which suggest that the model has a good generalization performance.

|  | **Slope** | **Intercept** | **PCC (train)** | **PCC (test)** | **NRMSE% (train)** | **NRMSE% (test)** |
| --- | --- | --- | --- | --- | --- | --- |
| **MVE** | 0.90 | 1.44 | 0.85 | 0.76 | 11.65 | 17.06 |
| **EV1** | 1.05 | 0.68 | 0.83 | 0.77 | 13.55 | 18.58 |
| **EV1/MVE** | 1.05 | -8.69 | 0.86 | 0.94 | 13.44 | 12.79 |
| **MEF** | 1.95 | 7.01 | 0.80 | 0.76 | 16.77 | 18.12 |
| **EF25-75%** | 1.04 | 0.33 | 0.79 | 0.80 | 17.21 | 14.81 |

**Supplementary** **Table S3: Anthropometric and demographic information of all 9 subjects who underwent the novel guided breathing paradigm in Figure 2B.**

| ***Subject*** | ***Gender*** | ***Age*** | ***Place of Origin*** | ***Weight (kg)*** | ***Height (cm)*** | ***BMI (kg/m^2^)*** | ***Chest circumference (cm)*** | ***Predicted FVC (L)*** |
| --- | --- | --- | --- | --- | --- | --- | --- | --- |
| GBDD-01 | Male | 24 | Malaysia | 77 | 179 | 24.03 | 89 | 5.34 |
| GBDD-02 | Male | 23 | Pakistan | 67 | 172 | 22.65 | 80 | 4.92 |
| GBDD-03 | Female | 21 | Hong Kong | 52.4 | 156 | 21.53 | 69 | 3.34 |
| GBDD-04 | Female | 18 | Indonesia | 51 | 154 | 21.50 | 74 | 3.26 |
| GBDD-05 | Male | 30 | Tunisia | 85 | 180 | 26.23 | 97 | 5.30 |
| GBDD-06 | Male | 30 | Hong Kong | 74 | 181 | 22.59 | 82 | 5.36 |
| GBDD-07 | Male | 21 | Taiwan | 64 | 180 | 19.75 | 83 | 5.45 |
| GBDD-08 | Male | 27 | Hong Kong | 60 | 172 | 20.28 | 82 | 4.85 |
| GBDD-09 | Male | 24 | Hong Kong | 64 | 169 | 22.41 | 81 | 4.72 |

**Supplementary Table S4. Anthropometric, demographic and clinical information of the subjects in the COVID-19 case study.** Subject GB-01 is the COVID-19 discharged subject, whereas subjects GB-02 and GB-03 are the age- and gender-matched healthy controls. Note that all subjects do not have existing heart or lung conditions.

| ***Subject*** | ***Gender*** | ***Age*** | ***Place of Origin*** | ***Weight (kg)*** | ***Height (cm)*** | ***BMI (kg/m^2^)*** | ***Chest circumference (cm)*** | ***Predicted FVC (L)*** | ***Date tested Positive*** | ***Symptoms*** | ***Date of Hospital Admission*** | ***Date of Hospital Discharge*** | ***Date of Recovery*** | ***Number of cigarettes smoked per week*** | ***Other lung and heart diseases*** |
| --- | --- | --- | --- | --- | --- | --- | --- | --- | --- | --- | --- | --- | --- | --- | --- |
| GB-01 | Male | 33 | Hong Kong | 79.5 | 186 | 22.98 | 90 | 5.63 | 20/12/2020 | Fever; Coughing | 20/12/2020 | 24/12/2020 | 13/01/2021 | 0 | N/A |
| GB-02 | Male | 24 | Malaysia | 77 | 179 | 24.03 | 89 | 5.34 | N/A | N/A | N/A | N/A | N/A | 0 | N/A |
| GB-03 | Male | 28 | France | 58 | 178 | 18.31 | 81 | 5.21 | N/A | N/A | N/A | N/A | N/A | 0 | N/A |

**Supplementary Table S5: Evaluation of** **the error induced by varying the belt from the normal position, varying the subject’s posture from the standing position, and repeating the test on a different day.** The normalized root mean square error is evaluated for different belt position, subject’s posture and day while the regression model for the prediction of the spirometry indicators is trained based on the measurement acquired with the belt at the instructed position, at the standing posture, and day 1, respectively. The errors induced by varying the belt 1 cm above to 5 cm below are comparable to the ones obtained from the normal position, while the errors when the belt is 5 cm below are the largest. Similarly, the errors induced by varying the subject’s posture from standing, sitting to laying are comparable, while the errors when the subject is laying are the largest. Finally, the errors when the test is repeated on day 11 are a slightly larger than day 1.

| **Regression model** | **Belt position \| Subject's posture** | **NRMSE% in MVE** | **NRMSE% in EV1** | **NRMSE% in EV1/MVE** | **NRMSE% in MEF** | **NRMSE% in EF25-75%** |
| --- | --- | --- | --- | --- | --- | --- |
| **Trained based on the instructed position** | **1cm above** | 14.2 | 5.56 | 7.88 | 8.99 | 9.12 |
|  | **2cm below** | 12.57 | 17.8 | 24.94 | 16.28 | 16.33 |
|  | **5cm below** | 17.74 | 13.96 | 28.4 | 18.01 | 18.62 |
|  | **Instructed** | 16.6 | 7.51 | 20.81 | 11.54 | 13.79 |
| **Trained based on the standing posture** | **Laying** | 19.45 | 12.79 | 8.79 | 15.35 | 12.81 |
|  | **Sitting** | 9.72 | 10.09 | 11.13 | 11.36 | 12.4 |
|  | **Standing** | 16.6 | 7.51 | 20.81 | 11.54 | 13.79 |
| **Trained based on day 1** | **Day 1** | 15.06 | 17.78 | 8.91 | 15.5 | 11.53 |
|  | **Day 11** | 18.86 | 14.86 | 23.2 | 20.56 | 26.75 |
