# Supplementary material for: Affordable, portable and self-administrable electrical impedance tomography enables global and regional lung function assessment
Source: Sci Rep. 2022 Nov 30;12:20613. doi: 10.1038/s41598-022-24330-2 (PMC9712422; doi:10.1038/s41598-022-24330-2)
Supplement: Supplementary file 2 — Supplementary Information 2. [file 41598_2022_24330_MOESM2_ESM.docx]

# Online methods

## Clinical trial registration

This study was registered on ClinicalTrials.gov (NCT05346081) on 26^th^ April 2022.

## Ethics

The study protocols and informed consent forms were reviewed and approved by a centralized Institutional Review Board (Hong Kong West Cluster IRB) Protocol Number: (IRB/REC No. UW 21-644) on 29 September 2021 and the Human Research Ethics Committee (HREC) of The University of Hong Kong on 9 February 2021. All methods were performed in accordance with the relevant guidelines and regulations and the reporting of the results follows the guidelines of strengthening the reporting of observational studies in epidemiology (STROBE)^1^. Informed consent was obtained from all subjects who participated in the study.

## Hardware

The hardware consists of an electrode lung belt for emitting and receiving electrical signals, and a portable EIT console (**Figure 1A**). The electrode belt consists of a silicone disinfectable band with sixteen equally spaced carbon gel electrodes (BJD-A, Bestpad, Shenzhen, China). The elastic electrode belts come in sizes ranging from 65 cm – 120 cm in length with extendable range of 10% of its original length. Sixteen 4 x 4 cm^2^ electrodes are used for belts longer than or equal to 75 cm, while the same electrodes are trimmed to 3 x 4 cm^2^ for belts shorter than 75 cm to avoid contact between adjacent electrodes.

The portable EIT console consists of five modules (**Supplementary Figure S1**). A power and battery management module provides constant power supply to all other electronic modules through the power socket or the Li-ion battery. A current generation module primarily includes a digitally programmable analog sine wave generator and a current generator successively to generate an alternating current (a.c) of 1 mApp and a voltage amplitude of 1 Vpp. A lowpass filter is used to suppress total harmonic distortion and ambient electromagnetic interference (e.g., power line noise). A signal distribution and readout module introduce the generated current to the subject via the 16-electrode belt using a set of CMOS multiplexers (MUXs). Four MUXs are used, in which two MUXs are employed for current injection and the other two for voltage readout. The MUXs are configured into the adjacent-scan pattern through the microcontroller unit (MCU). A data acquisition module is the analog front-end (AFE) that acquires, measures, and amplifies the differential voltage from the electrodes. The AFE comprises a four-stage wide input differential amplifier with high common-mode rejection ratio (CMRR), and a bandpass filter. A control and output module consists of an analog-to-digital converter (ADC), an MCU and a wireless communication chip. The differential voltage obtained from the data acquisition module are digitized by three 12-bit ADCs using triple interleaved ADC method to achieve higher sampling rate and higher signal-to-noise ratio (SNR), processed in the MCU unit, and transferred to the cloud server for image reconstruction and processing. The use of triple interleaved ADC method has enabled us to achieve flexible frame rate up to 50 frame per second (fps), which is higher than typical systems in the market and literature^2^. The higher frame rate allows better detection of high frequency changes in conductivity, such as the onset of forced exhalation during spirometry tests. The high frame rate can also be used to trade for higher signal-to-noise ratios through averaging and other signal or/and image processing methods. In this study, we applied a stimulation frequency of 35KHz and for best performance we have taken 1000 samples per measurement at a raw data (potential difference) sampling frequency of 7.2MHz. The alternating current (ac) was injected sequentially between all adjacent electrode pairs and the potential differences were measured across other thirteen adjacent electrode pairs, making a total of 208 (16 × 13) differential voltage measurement per frame. Although a single ADC in STM32F405 has a maximum clock frequency of 2.4MHz, we have utilized all 3 ADCs in the STM32F405 MCU and applied the triple interleave method. This allowed us to achieve a raw data sampling frequency to 7.2MHz. Hence, 7.2MHz / 1000 measurements / (16 ×13) electrode pair measurements per cycle approximates 33 fps. We can increase the fps by reducing the number of samples per measurement, for example for 50 fps we have to take around 700 samples per measurement. The observed SNR ranged from 45-55 dB.

## Breathing paradigms

We defined two breathing paradigms, a forced breathing paradigm and a guided breathing paradigm. The subjects performed all breathing modes in the upright position. For the forced breathing paradigm, the subjects were guided by the curves shown in **Figure 1B** to perform three shallow breaths at a rate of eighteen cycles per minute, followed by an inhalation to their maximum (or mid) lung capacity. Subsequently, they were instructed to exhale fast and abruptly (or slowly) during an exhalation segment lasting for 6 seconds. At the end of the exhalation phase, the subjects were guided to inhale again to their maximum (or mid) lung capacity, followed by an exhalation to their normal breathing exhalation volume, and lastly another three guided shallow breaths at a rate of eighteen cycles per minute. The full paradigm lasted 26 seconds. The middle section of the inhale and exhale had four variants resulting in four types of breathing efforts which we denoted as full inhale and fast exhale, full inhale and slow exhale, mid inhale and fast exhale, and mid inhale and slow exhale. At the same time, the subjects were instructed to breathe into and out of the spirometry device (Spirobank Smart, Medical International Research, Italy) via a mouthpiece to acquire the spirometry indicators and the volume-time curves during the forced exhalation phase. For the guided breathing paradigm, the subjects were instructed to follow the breathing instructions on a screen for 60 seconds as shown in **Figure 5A**, which consisted of regular inhale-and-exhale cycles at a constant rate of twelve breaths per minute. The choice of a relatively slow breathing rate (typical range for healthy adult 12-20bpm^3^) is made in order to impose a certain degree of breathing challenge to the users. That is a normal subject would tend to breathe a larger amount of air at each cycle during this exercise.

## Subjects

Fourteen subjects performed around twenty repetitions of forced breathing paradigm (**Figure 1B-C**), nine subjects performed around four repetitions of guided breathing with deep and shallow breathing modes (**Figure 5B**), and a COVID-19 discharged subject and two age- and gender-matched healthy controls performed guided breathing across a span of 10 days (**Figure 6**). We have attempted to recruit and acquire data from as many subjects as possible who satisfied the inclusion criteria (**supplementary note 1**), during the period from January 2021 and December 2021. Note the COVID-19 discharged subject conducted the tests by himself with a similar but separate system. The current generation module of this system included a 12-bit digital-to-analog converter (DAC) and a current generator successively to generate an alternating current (ac) of amplitude 1-2 mApp. The AFE, comprised two non-inverting amplifiers and two 12-bit ADC for signal acquisition. The anthropometrics of all recruited subjects are summarized in **Supplementary Tables S1, S3 and S4**.

All subjects in this work, unless otherwise stated, performed the tests in standing position and placed the belt at the thorax around the T4 and T5 vertebrae, that is right below the nipples for man and right below the breast for females. To ensure high data quality, subjects were advised to follow the guide to wear the belt that is given through the mobile app (**Supplementary** **guide to wear the belt**). The gel electrodes were replaced every time before each usage and the test lasted a maximum of one hour duration.

For the forced breathing paradigm, subjects performed variable efforts to simulate different forced vital capacity (FVC), forced expiration volume in 1 second (FEV1), FEV1/FVC ratio, peak expiratory flow (PEF) and forced expiratory flow at 25–75% of forced vital capacity (FEF25–75%). From here-on the simulated spirometry indicators are referred to as maximal volume engaged (MVE), exhaled volume in 1 second (EV1), EV1/MVE ratio, maximum expiratory flow (MEF), and expiratory flow at 25–75% of maximum volume engaged (EF25-75%), corresponding to FVC, FEV1, FEV1/FVC, PEF, FEF25-75% respectively. These subjects followed four distinct types of instructions and performed five repetitions for each type of instruction. The first type is the spirometry-like forced breathing paradigm, i.e., inhale to full capacity, and exhale as fast as possible. This is to obtain the largest possible values of MVE, EV1, EV1/MVE, MEF and EF25-75%. The second type is to inhale to the full capacity but exhale slowly. This is to simulate a low EV1, EV1/MVE, MEF and EF25-75%, while MVE remains high. The third type is to inhale to mid capacity, and exhale as fast as possible. This is to simulate a low MVE, and a high EV1/MVE. The fourth type is to inhale to mid capacity and exhale slowly. This is to simulate a low MVE, EV1, EV1/MVE, MEF and EF25-75%.

## Image reconstruction and processing

The reference voltage data frame for time-difference EIT image reconstruction is set to the mean data frame across the full time-series after gain normalization. Individual data frames are denoised by setting outlier voltage values above and below system thresholds to the corresponding value in the reference data frame. The EIT images are reconstructed from the denoised data frames and reference voltage data frame using one-step linear Gauss-Newton solver^4^, with a regularization matrix based on the Newton's one-step error reconstructor (NOSER) prior with p = 0.35 and a regularization hyperparameter of λ^2^ = 0.005.

As the sampling rate is not strictly consistent during data acquisition, the initially reconstructed time-series EIT images are interpolated into a new time-grid of regular sampling rate, resulting in a new series of timestamp-corrected EIT images. Further spatiotemporal filtering is applied on every 3D spatiotemporal image series to reduce temporal noise and spatial artifacts. For the forced breathing paradigm, a 0.8s-wide moving average is used to filter the temporal waveforms at each voxel. For the guided breathing paradigm, a 3rd order Butterworth filter of 0.083 – 0.5 Hz passband was used to filter out the irrelevant signals embedded in temporal waveforms at each voxel, such as the cardiac related signals (i.e., 60 – 80 beats per minute), but to keep breathing related signals (i.e., 5 – 30 breaths per minute). The filtered image series is then transformed from corresponding initial triangular simplices into a 64×64-voxel rectangular grid by using interpolation weights defined by a sigmoid function.

For each time-series of images, a global conductivity curve, an amplitude map and a correlation map are calculated. The global conductivity curve represents the dominant conductivity signal due to breathing and is obtained by a weighted sum of the conductivity curves at all voxels. The amplitude map represents the conductivity variation at different voxels. The time series at each voxel is first partitioned into segments of duration T_d_ seconds and a stride of T_s_ seconds. For forced breathing, T_d_=6s and T_s_=6s, whereas for guided breathing T_d_=15s and T_s_=3s. The amplitude of the corresponding voxel is evaluated as the 50^th^ and 100^th^ percentile of the maximum change of conductivity at all time-segments for guided breathing and forced breathing, respectively. The correlation map represents the voxel-wise correlation between the global conductivity-time curve and the respective conductivity-time curve.

## Data exclusion

Trials were excluded by inspecting the correlation map and the global conductivity curve. The accepted trials showed two positively correlated clusters in the correlation map (corresponding to two lung regions) and a global conductivity curve which followed the instructed curve. Upon data rejection, the number of trials remaining for the forced breathing paradigm is 285 from twelve subjects, for the COVID-19 case study is 21 from three subjects, while no trials are rejected for the guided breathing paradigm with deep and shallow breaths.

## Mapping between conductivity and volume

For the forced breathing paradigm, a functional mapping to predict the volume-time curve ($V(t))$ from the conductivity-time curve ($\gamma\left( t \right)$) and anthropometrics (including chest circumference ($CC$), weight ($W$), height ($H$), weight/height ($W/H$), gender and age) is learnt from a sub-set (training set) of the collected data, then evaluated with the remaining sub-set (test set). The test-set is obtained by excluding all data from two randomly chosen participants and another 10% randomly chosen data from the remaining participants. The training-set is the remaining data from the global set after excluding the test-set. The proportion of the test-set is 24%. This splitting strategy is adopted to verify whether the trained model is capable to generalize unseen data and subjects with different anthropometrics.

Since, the volume-time curve is measured only during the forced exhale, we first extracted the conductivity curve corresponding to the forced exhale and inhale is by excluding the first and last segments corresponding to the guided shallow breathing. The starting time of the forced exhale is obtained using the back-extrapolation method as in standard spirometry data analysis^5^.

Inspection of the scatter plots between the conductivity and the volume for different subjects showed a high degree of correlation (**Figure 1C and Figure 2A**). However, the slopes of the best fit lines for each subject data are different, see **Figure 2B.** The correlation coefficients between the slope and anthropometrics (**Figure 2C**) demonstrate that the slope is highly and positively correlated with the weight, weight-height ratio (W/H), and chest circumference. In order to train a model with anthropometric-dependent slope, we trained a linear model with the following dependent variables ($\gamma\left( t \right), CC\times\gamma\left( t \right), \left( W/H \right)\times\gamma\left( t \right), \left( W/H \right),\mathrm{and} CC$), the coefficients obtained using regression analysis lead to the following relation

$$V\left( t \right)=\left[ 0.909\times\left( W/H \right)+0.014\times CC-1.236 \right]\times\gamma\left( t \right)+10.24\times W/H-0.065\times CC$$

$+2.184$.

All regression coefficients were significant (p < 0.05; two-sided t-test), demonstrating the importance of all variables in the volume estimation. These findings are consistent with the previous study that explores the relationship between the change in lung volume and EIT measurement as they are indicative of the total volume of the conductive medium under inspection as well as the distribution of the body fat^6^. The PCC between the predicted volume and the measured volume is 0.89 for the training set and 0.8 for the testing set. The normalized root mean squared error (NRMSE) of the predicted volume is 10.4% and 13.4% for the training and testing set, respectively. This shows that the developed regression model can predict the volume from the conductivity and anthropometrics for a wide dynamic range and for different subjects of different anthropometrics.

## Spirometry indicators

The EIT indicators and functional maps corresponding to MVE, EV1, MVE/EV1, MEF and EF25-75% are calculated from the estimated global volume curve and the voxel-wise conductivity curve as follows^5^: (1) MVE is obtained by the difference between the maximum and minimum volume/conductivity changes; (2) EV1is obtained by the difference between the volume/conductivity change at the starting time of the exhalation and one second after; (3) EV1/MVE is the ratio of EV1 and MVE; (4) MEF is the maximum value of the time derivative of the volume/conductivity curve; and (5) EF25-75% is the average flow during the expiration from 25% of MVE to 75% of MVE.

Further linear correction (of the form: Indicator(corrected) = slope×Indicator(computed) + intercept) was needed to achieve better estimation of the global spirometry indicators (**Figure 3B**). The slope, intercept, and the evaluation metrics (PCC and NRMSE% for both training and testing set) are shown in **Supplementary Table S2**. Overall, the PCC between the predicted and measured indicators is larger than 0.75 and the NRMSE% is smaller than 18.5%.

## Regional analysis

Regions of interest (ROIs) are defined using a threshold-based segmentation of the amplitude-correlation product map. The largest cluster with the most selected voxels localized in the left-half and right-half of the product image are considered as the left and right lung clusters, respectively. For forced breathing, a group threshold was applied onto all repetitions performed by the same subject in a single trial. The group threshold value was determined by finding the mean of threshold values which selected the top 35% voxels with the largest amplitude-correlation product across repetitions of the fourth (weakest) breathing effort. This thresholding strategy is used to compare the activated voxels for different breathing effort. For guided breathing, individual thresholds were applied to each repetition to select the top 35% voxels with the largest amplitude-correlation product. The lung clusters were further divided into four ROIs, namely the anterior left, posterior left, anterior right and posterior right lungs (**Figure 4**). The anterior-posterior division was defined by the horizontal line passing through the midpoint between the topmost and bottommost voxels in both lung clusters.

The left and right conductivity waveforms (**Figure 2B**) are obtained by averaging the temporal signals of voxels within both lung clusters. The frequency spectra of the left-right waveforms are obtained by computing their fast Fourier transforms. Regional EIT indicators including the number of activated voxels, total amplitude, and the coefficient of variation (C.V.) shown in **Figure 2B-E** are computed from amplitude image. The number of activated voxels is the count of voxels in the corresponding ROI. The total amplitude is the sum of the voxel amplitude within different ROIs. The C.V. is the ratio of the standard deviation over mean in the top 75% voxels within each ROI. C.V. were compensated for potential system-dependent SNR. C.V. serves to monitor homogeneity of amplitudes as a significant consistent loss in homogeneity could imply decreased lung function. For the forced breathing paradigm, additional regional indicators (MVE, EV1, EV1/MVE ratio, MEF and EF25-75%) were computed for each lung ROI, by averaging the corresponding functional indicator maps within each ROI (**Figure 4**).

## Comparison of functional maps and regional indicators

All functional maps were masked with the lung clusters to remove irrelevant values in non-lung voxels. At each breathing effort, the average normalized functional maps across subjects are obtained (**Figure 4B**). Voxels less than 0.1 were additionally masked in the maps to elucidate the relative sizes of the lung clusters. The mean functional indicators in each ROI were used to predict the spirometry indicators at each region. A mapping function was obtained by training a multiple linear regression model for each indicator with the input features used in the previous section but using EIT indicators computed from the average waveform within the lung clusters (instead of the extracted global waveform). The trained models were then used to compute the predicted spirometry indicators at each ROI (**Figure 4C**). Two-way ANOVA followed by Bonferroni multiple comparisons was applied to compare the predicted regional indicators across breathing efforts and ROIs.

For each guided breathing paradigm, the amplitude maps were masked with the lung clusters. For shallow and deep guided breathing, the amplitude maps were normalized by the maximum voxel amplitude across all repetitions performed. The average amplitude maps at shallow and deep breathing are obtained by computing the mean normalized amplitude maps at each breathing depth (**Figure 5B**). For the COVID-19 case study, each amplitude map was normalized by its maximum voxel amplitude, and the average amplitude maps were obtained by averaging across different subject groups (patient and healthy controls) (**Figure 2C**). Two-way ANOVA followed by Bonferroni multiple comparisons was applied to compare the number of activated voxels and total amplitude between the left and right lungs across breathing depth or patient/control group (**Figure 5B and Figure 6A**). For shallow and deep guided breathing, the regional waveforms and frequency spectra were normalized by their maximum value across all trials, and the mean normalized breathing waveforms and frequency spectra were presented with ± SEM (**Figure 5B**). For the COVID-19 case study, the mean left and right C.V. in the patient and healthy control groups were presented (**Figure 6A**). Two-way ANOVA followed by Bonferroni multiple comparisons was applied to compare the left and right lung C.V. between subject groups. To show the trends in C.V. across time, linear regression models were fit for the left and right lung C.V. against day of data collection for each subject (**Figure 6B**). For the COVID-19 discharged subject, the C.V. against time in each ROI were also fit with linear regression models to further localize the reduction in C.V. across the period of recovery (**Figure 6C**).

## References

1. Vandenbroucke, J. P. Strengthening the Reporting of Observational Studies in Epidemiology (STROBE): Explanation and Elaboration. *Ann Intern Med* **147**, W (2007).

2. Hong, S., Lee, J., Bae, J. & Yoo, H.-J. A 10.4 mW electrical impedance tomography SoC for portable real-time lung ventilation monitoring system. *IEEE Journal of Solid-State Circuits* **50**, 2501–2512 (2015).

3. Barret, K. E., Boitano, S. & Barman, S. M. *Ganong’s review of medical physiology*. (McGraw-Hill Medical, 2012).

4. Liu, B. *et al.* pyEIT: A python based framework for Electrical Impedance Tomography. *SoftwareX* **7**, 304–308 (2018).

5. Miller, M. R. ATS/ERS task force: standardisation of spirometry. *Eur Respir J* **26**, 319–338 (2005).

6. Coulombe, N., Gagnon, H., Marquis, F., Skrobik, Y. & Guardo, R. A parametric model of the relationship between EIT and total lung volume. *Physiological measurement* **26**, 401 (2005).
