# Supplementary material for: Affordable, portable and self-administrable electrical impedance tomography enables global and regional lung function assessment
Source: Sci Rep. 2022 Nov 30;12:20613. doi: 10.1038/s41598-022-24330-2 (PMC9712422; doi:10.1038/s41598-022-24330-2)
Supplement: Supplementary file 3 — Supplementary Information 3. [file 41598_2022_24330_MOESM3_ESM.pdf]

Device setup (mobile)

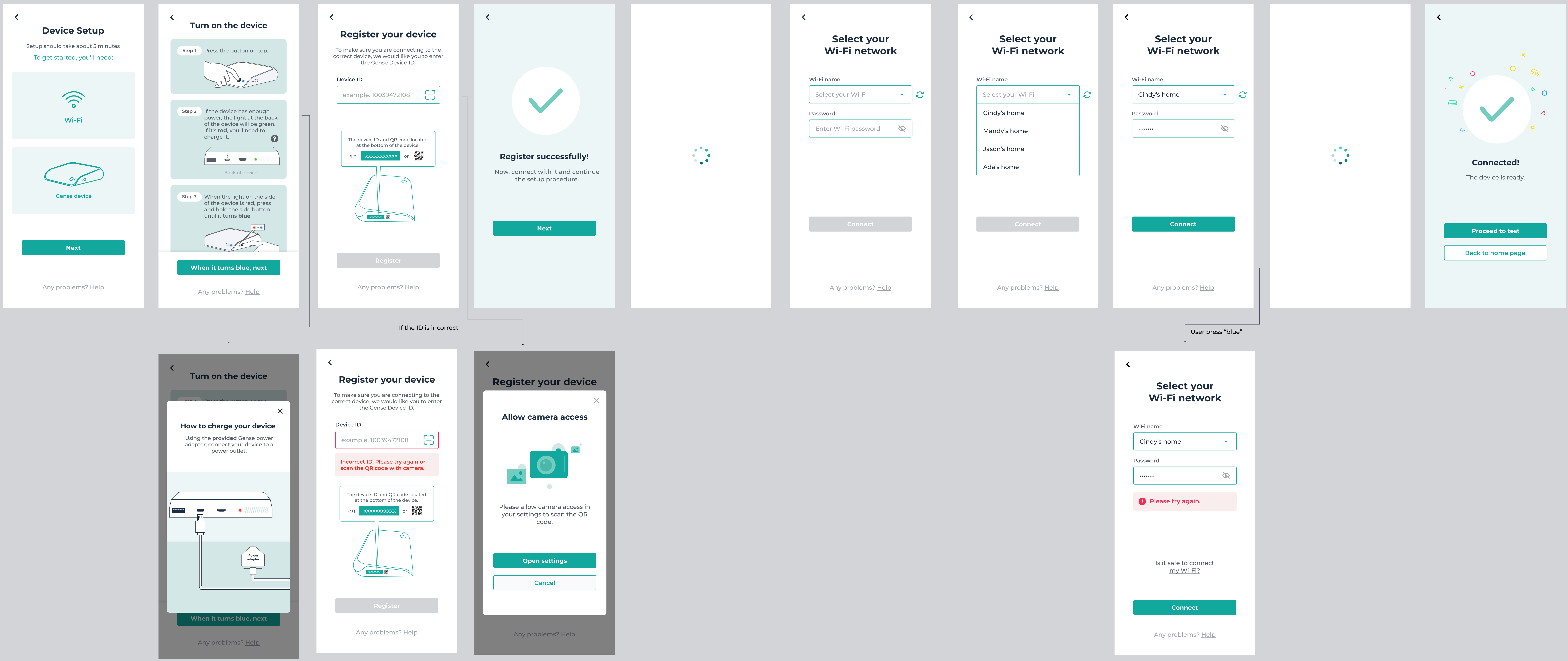

Step 1:
Press the button on top of the device to turn on the device. The LED on top should turn blue. Wait for about a minute until the LED on the side of the device turns red

Step 2:
When the LED on the side of the device is red, press and hold the side button until the side LED turns from red to blue
