# Supplementary figures and images for: Affordable, portable and self-administrable electrical impedance tomography enables global and regional lung function assessment
Source: Sci Rep. 2022 Nov 30;12:20613. doi: 10.1038/s41598-022-24330-2 (PMC9712422; doi:10.1038/s41598-022-24330-2)

# Guide to perform lung test

## Guided breathing test

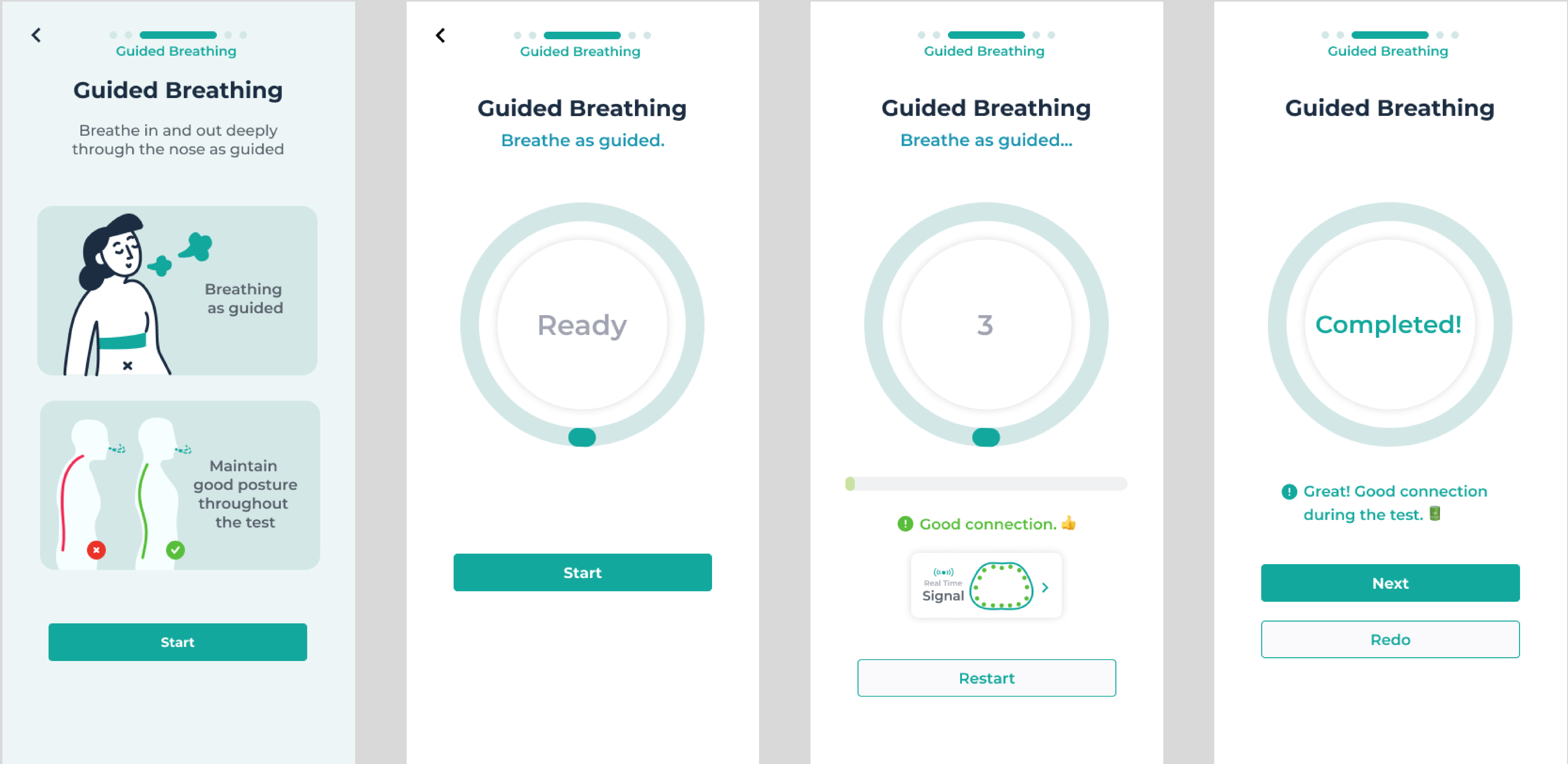

## Spiromrtry test

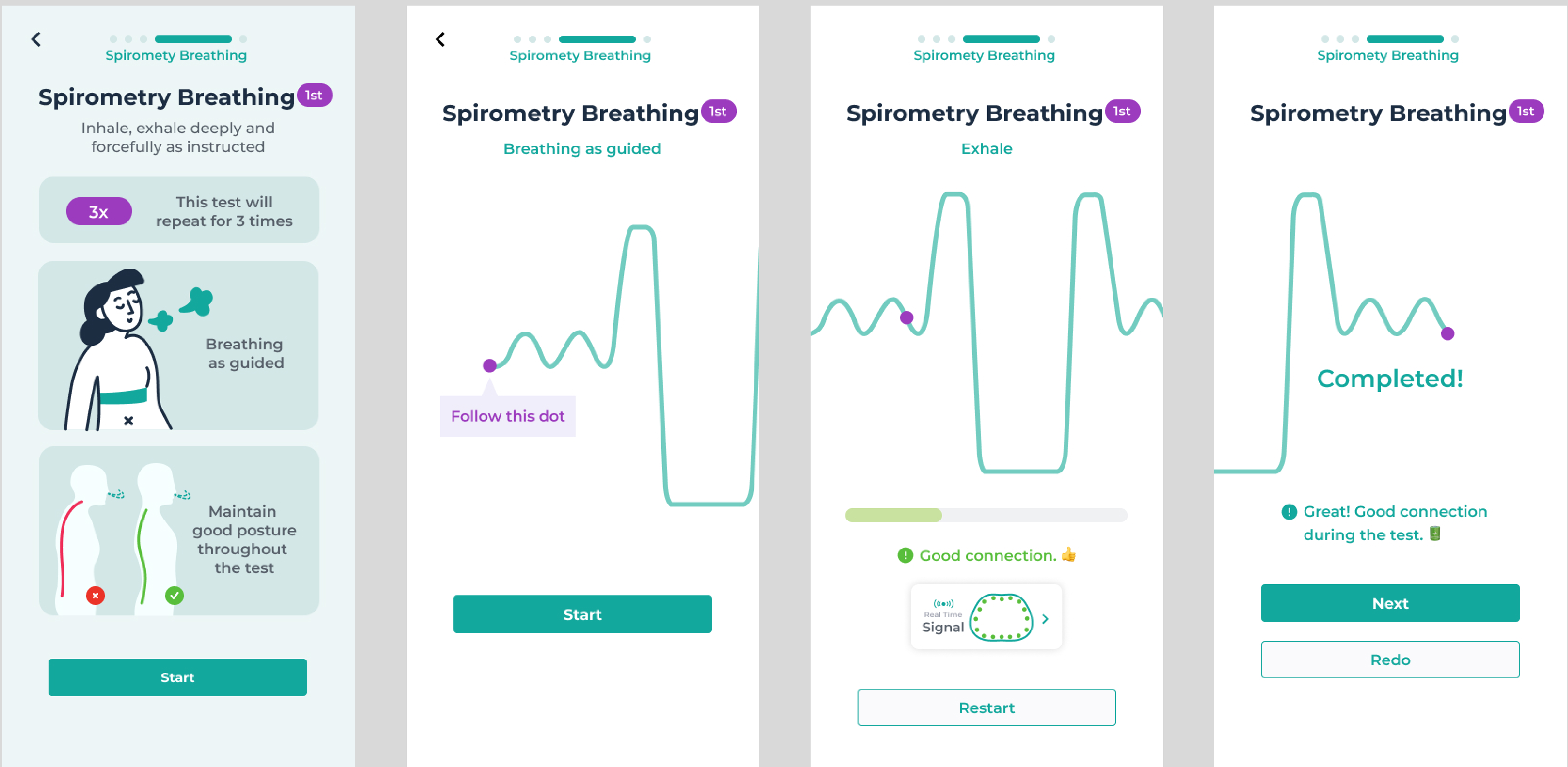

Supplement: Supplementary file 4 — Supplementary Information 4. [file 41598_2022_24330_MOESM4_ESM.pdf]
