# Supplementary material for: Affordable, portable and self-administrable electrical impedance tomography enables global and regional lung function assessment
Source: Sci Rep. 2022 Nov 30;12:20613. doi: 10.1038/s41598-022-24330-2 (PMC9712422; doi:10.1038/s41598-022-24330-2)

Lung Guide to wear the belt

Do Test Alone (Male user)

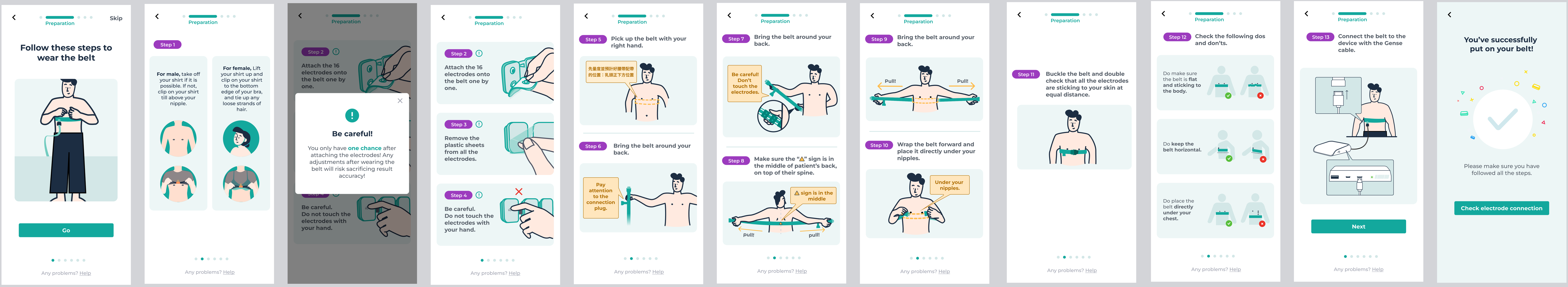

Do Test with Helper (Male user)

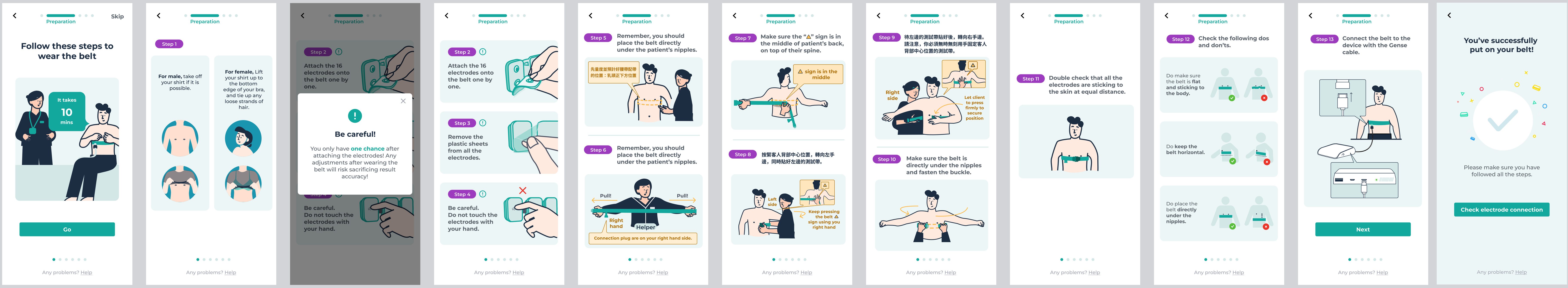

Supplement: Supplementary file 5 — Supplementary Information 5. [file 41598_2022_24330_MOESM5_ESM.pdf]
